# Expanded carrier screening in Chinese patients seeking the help of assisted reproductive technology

**DOI:** 10.1002/mgg3.1340

**Published:** 2020-06-23

**Authors:** Yanping Xi, Guangquan Chen, Caixia Lei, Junping Wu, Shuo Zhang, Min Xiao, Wenbi Zhang, Yueping Zhang, Xiaoxi Sun

**Affiliations:** ^1^ Shanghai Ji Ai Genetics & IVF Institute Obstetrics and Gynecology Hospital of Fudan University Shanghai China; ^2^ WuXi NextCODE Genomics (Shanghai) Co., Ltd. Shanghai China; ^3^ Key Laboratory of Female Reproductive Endocrine Related Diseases Obstetrics, and Gynecology Hospital of Fudan University Shanghai China

**Keywords:** assisted reproductive technology, expanded carrier screening, Han Chinese ethnicity, preimplantation genetic testing, recessive disease

## Abstract

**Background:**

Expanded carrier screening (ECS) has emerged as an effective approach to identify at‐risk couples (ARCs)—before they initiate attempts at reproduction—who possess a high probability of having a child affected by severe recessive diseases. The objective of this study was to evaluate the clinical utility of ECS in Chinese patients seeking the help of assisted reproductive technology (ART).

**Methods:**

An ECS test, which covers 201 genes implicated in 135 recessive (autosomal or X‐linked) diseases, was routinely offered to all ART patients in a single genetics and in vitro fertilization clinic. Additional options for preimplantation or prenatal genetic diagnosis were discussed and offered to all ARCs. All ECS results were aggregated and the clinical decisions of the ARCs were surveyed.

**Results:**

A total of 2,923 ART patients, representing 1,462 couples, were screened. Overall, 46.73% of the individuals were found to be the carriers for at least 1 of the 135 diseases. Of the tested couples, 2.26% (*n* = 33) were identified as ARCs. As of the completion of this study, 21 (63.6%) ARCs have decided to avert an affected pregnancy with the help of preimplantation genetic testing for monogenetic conditions. The cumulative carrier rate of the 187 autosomal recessive genes in the ECS panel for the 2,836 Han Chinese individuals without a family history was estimated to be 45.91%. The estimated at‐risk couple rate indicates that the screening for only the top 31 genes with gene carrier rates >0.5% would identify more than 94% of the ARCs identified by screening all 187 genes.

**Conclusion:**

Our study demonstrates that ESC yields a significant clinical value for ART patients in China. In addition, by estimating the yields of the ECS panel, we identify genes that are appropriate for screening the Han population.

## Graphical abstract

We launched a pilot population based expanded carrier screening(ECS) for 135 severe recessive Mendelian conditions in Chinese patients seeking the help of assisted reproductive technology (ART). Our study demonstrates that ESC yields a significant clinical value for the ART patients in China. In addition, by estimating the yields of the ECS panel, we identify genes that are appropriate for screening the Han population.

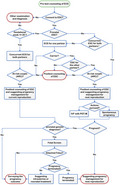

## 1 INTRODUCTION

Currently, assisted reproductive technology (ART), especially in vitro fertilization (IVF), is the most effective method for infertile couples to achieve pregnancy (Qiao & Feng, 2014; Treff & Zimmerman, 2017). Infertility affects 1 in 6 couples of reproductive age (Treff & Zimmerman, 2017). Within the framework of IVF, oocytes and/or embryos can be tested by preimplantation genetic testing (PGT) to prevent embryos with genetic disorders from being implanted (Kuliev & Rechitsky, 2017). This not only allows for the establishment of pregnancies, but for the birth of genetically healthy babies (Kuliev & Rechitsky, 2017; Martin et al., 2015; Qiao & Feng, 2014; Treff & Zimmerman, 2017). Since the first baby screened by PGT for X‐linked diseases was delivered in 1990 (Handyside, Kontogianni, Hardy, & Winston, 1990), PGT has evolved into an established clinical procedure in reproductive and genetic medicine (Kuliev & Rechitsky, 2017; Lee, Chow, Yeung, & Ho, 2017). Previous data illustrate that the technology is safe and reliable with no significant adverse effects (Kuliev, 2012; Kuliev & Rechitsky, 2017; Liebaers et al., 2010; Rechitsky et al., 2015, 2016; Treff & Zimmerman, 2017).

As a component of PGT, preimplantation genetic testing for monogenic conditions (PGT‐M) is designed mainly to test for single‐gene disorders (Imudia & Plosker, 2016) and has been performed for over 400 different genetic disorders (Kuliev & Rechitsky, 2017). Simultaneous testing for chromosomal aneuploidy and translocations is also possible from a single biopsy obtained for PGT‐M (Kuliev & Rechitsky, 2017; Treff & Zimmerman, 2017). With the development of PGT‐M, the demand for the identification of couples at the risk of conceiving children with recessive disorders is dramatically increasing. At‐risk couples (ARCs) are those in which both partners carry pathogenic (P) or likely pathogenic (LP) variants in the same gene, or female carries an X‐linked P or LP variant. These couples are at high risk of giving birth to offspring with severe genetic diseases. Previously, ARCs were often identified by genetic diagnosis following the birth of an affected child. In recent decades, however, carrier screening has emerged as an alternative approach that identifies ARCs before they initiate attempts at reproduction (Edwards et al., 2015; Franasiak et al., 2016; Johansen Tab er et al., 2019; Kuliev & Rechitsky, 2017; Martin et al., 2015; Treff & Zimmerman, 2017).

Carrier screening aims to identify healthy individuals with a heterozygous deleterious variant of a recessive (autosomal or X‐linked) disorder. It was first introduced in the 1970s as a means to detect the likelihood of inherited conditions (Stamatoyannopoulos, Motulsky, & Ebling, 1974). Initially, carrier screening programs were established within specific ethnic groups who had a very high prevalence of certain conditions, such as ancestry‐based screening for Tay–Sachs disease in Ashkenazi Jewish communities (Kaback, 2000). Later, the cystic fibrosis (CF) screening became available after CF‐associated genes were identified (Riordan et al., 1989). After 2001, CF became the first disease recommended for pan‐ethnic routine carrier screening in the United States by several professional guidance associations, such as the American College of Obstetricians and Gynecologists (ACOG) and the American College of Medical Genetics (ACMG) (Obstetricians & Gynecologists, 2001, 2011). This promoted carrier screening as a more general practice for preconception and prenatal populations, tailored to specific conditions within ethnic groups. For instance, in some parts of the world, pan‐ethnic screening is used for hemoglobinopathies and thalassemia (Bajaj & Gross, 2014). More recently, carrier screening for spinal muscular atrophy (SMA) was suggested by the ACOG for all women considering pregnancy (or already pregnant), as well as additional screening based on the family history and ethnicity (Committee on, 2017; "Committee Opinion No. 691 Summary: Carrier Screening for Genetic Conditions," 2017; Prior, Professional, & Guidelines, 2008).

In recent decades, technological advances and decreases in the cost of sequencing have made expanded carrier screening (ECS) available and affordable. Next‐generation sequencing (NGS) has allowed ECS to evaluate hundreds of conditions in one test (Hallam et al., 2014; Nazareth, Lazarin, & Goldberg, 2015). Although many conditions are, themselves, rare, one study found that approximately 35% of individuals in their sample were carriers of at least one condition (Srinivasan et al., 2010). The author demonstrated that the screening for the most common genetic disease alone fails to identify most carriers in the general populations (Srinivasan et al., 2010). In addition, both cost efficiency and the conditions included in ECS tests have been widely discussed or studied (Beauchamp, Johansen Tab er, & Muzzey, 2019; Wilfond et al., 2018). Beauchamp et al studied the cost‐efficacy of a 176‐condition ECS and concluded that ECS can reduce the population burden of Mendelian disease in a cost‐effective manner when compared to many other common medical interventions (Beauchamp et al., 2019). Guo and Gregg suggested to guide the design of ECS panels with estimated carrier rates across genes (Guo & Gregg, 2019).

Although there have been quite a few studies on ECS, ranging from panel design (Bell et al., 2011; Martin et al., 2015) to clinical implementation (Franasiak et al., 2016), most of them are biased toward the people of European descent. The ECS data for China are quite limited. Sumin et al. recently performed ECS on 10,476 prenatal/preconception couples from 34 Chinese ethnic groups (Zhao et al., 2019). However, their study was limited in that it only tested for 12 genes associating with 11 Mendelian disorders. Moreover, it did not address the impact of ECS on the clinical decisions made by tested couples.

In the present paper, we applied an ECS panel of 202 genes implicated in 135 recessive diseases (121 autosomal recessive [AR] and 14 X‐linked) for ART patients in a local fertility center in China. Through this investigation, we aim to evaluate the clinical utility of ECS in Chinese ART patients.

## 2 METHODS

### 2.1 Ethical compliance

This study was approved by the ethics committee (institutional review board) of the Shanghai Ji Ai Genetics & IVF Institute (code JIAIE2019‐11).

### 2.2 Study design

ECS was routinely offered as an option to all patients seeking ART in a single genetics and IVF clinic between 1 May 2017 and 31 July 2019. Patients who elected to complete ECS were included in this analysis. Data management and tabulation were accomplished via self‐written Python and R scripts. Informed consent was obtained from each participant and the genomic data of individuals were de‐identified and analyzed in a cumulative manner.

### 2.3 Clinical principles and practice

**Figure 1:**
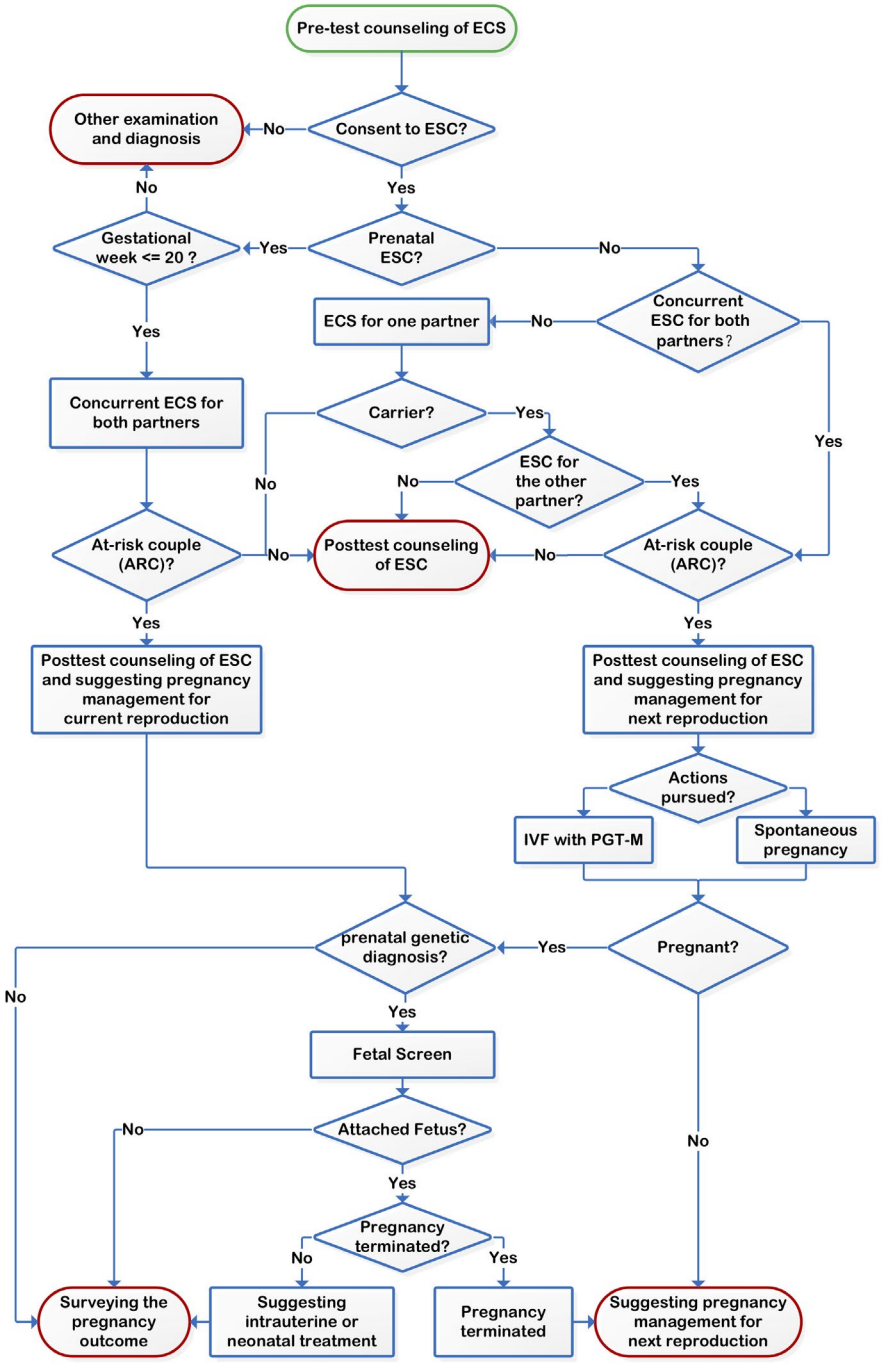
The ECS practice in the IVF clinic. The green ellipses represent the starting point of the process and the red ellipses represent an endpoint of the process. ECS, expanded carrier screening; IVF, in vitro fertilization

The clinical workflow of ECS in this study is illustrated in Figure 1. Pre‐test counseling for ECS was provided, which informed patients of the ECS program and discussed the risks, benefits, and limitations of screening. Patients who volunteered for ECS were divided into two groups according to whether they were pregnant or not. Women whose pregnancies were over 20 weeks of gestation were not recommended for ESC because it would be difficult to complete ESC as well as prenatal diagnosis before the pregnancy termination deadline of China (before 28 weeks of gestation). Spouses of pregnant women with gestational age less than 20 weeks were advised to undertake ECS concurrently in order to identify the couple's reproductive risk as early as possible. Individuals in non‐pregnant couples could choose to undertake ECS one at a time. If one partner of a couple was found to be a carrier for a specific condition, the other partner would be advised to undertake ESC as well. Post‐test counseling was provided to all ECS subjects to inform the couple of potential reproductive outcomes, as well as to inform couples of the residual risk of being a carrier even after receiving negative test results for each screened gene.

ESC results helped identify ARCs. In addition to the above‐mentioned post‐test genetic counseling, ARCs in which the female was already pregnant also received recommendations for prenatal genetic diagnosis (amniocentesis or chorionic villus sampling); otherwise, IVF with PGT‐M was recommended. For the patients who underwent PGT‐M, we applied the Illumina HumanKaryomap‐12 DNA Analysis Kit (Illumina, www.illumina.com) to identify unaffected embryos through linkage analysis. All pregnant women in the ARCs were advised to undertake prenatal genetic diagnosis whether through spontaneous pregnancy or IVF with PGT‐M. Those who failed to get pregnant were offered additional counseling, suggesting adjustments to pregnancy management for their next reproductive attempt.

Prenatal genetic diagnosis was performed for ARCs who volunteered following the ACOG guideline (Committee on & the Society for Maternal‐Fetal, 2016). At‐risk couples (ARCs) were allowed to voluntarily terminate or continue their pregnancy if the fetus was diagnosed as affected in prenatal genetic diagnosis. The former received advice on pregnancy management for their next reproductive attempt and the latter were recommended intrauterine or neonatal treatment if feasible. The pregnancy outcomes as well as birth defects of all children borne by ARCs were surveyed.

### 2.4 Disease selection and panel design

The ECS panel covers 201 genes implicated in 135 single‐gene recessive (AR or X‐linked) diseases (Table S1). These pathologic conditions were carefully chosen after considering ACMG recommendations (Edwards et al., 2015) and the perspective of many PGT couples in our clinic, who have a strong desire to reduce the medical burden of genetic diseases and improve the quality of life of future generations through screening and PGT. Broadly, we included severe childhood‐onset disorders with highly penetrant phenotypes, high‐prevalence monogenic diseases with moderate phenotypes, and disabilities that impact the quality of life for the entirety of the patient's life, such as severe hearing loss and blindness.

Single‐nucleotide variants (SNVs) and small indel variants located in exons and introns within 10bp‐regions of the selected genes were detected. The panel also detected an exon 7 deletion in *SMN1*(OMIM: 600354, reference sequence: NM_000344.3) for SMA and −α3.7, −α4.2, −SEA, −FIL, and −THAI variants for alpha‐thalassemia. Determining the relative distribution of two or more copies of*HBA1*(OMIM: 141800, reference sequence: NM_000558.4)and *HBA2*(OMIM: 141850, reference sequence: NM_000517.4) located in homologous chromosome 16 was beyond the capabilities of this analysis. P021 SMA and P140 HBA MLPA kits (MRC‐Holland) and capillary electrophoresis were used to verify the suspected positive variants. Quality control and data analysis were conducted using the Coffalyser.net software (MRC‐Holland, www.mlpa.com).

### 2.5 Genomic sequencing and data analysis

Exons of the 201 genes, along with their 10‐bp flanking intronic regions, in the subject's DNA were captured using an Agilent Custom Target Enrichment Probe Kit (Agilent). The DNA was then sequenced by high‐throughput sequencing on the Illumina HiSeq platform (Illumina, www.illumina.com). The resulting reads were mapped to the reference genome hg19 to identify the bases in all sequencing fragments. The sequencing coverage of each base was obtained from all genomic sequencing data. The Genome Analysis Toolkit (GATK) (McKenna et al., 2010) was used to detect SNVs, small indels, and the specific copy number variants mentioned above for *SMN1*, *HBA*, and *HBA2* genes.

### 2.6 Variant interpretation

The population‐based large‐scale sequencing databases gnomAD (Karczewski et al., 2020) was used to exclude mutations that occurred with high frequency in the normal population. The remaining variants were annotated with the Ensembl Variant Effect Predictor (VEP) (McLaren et al., 2010). The variants were classified according to the standards and guidelines issued by the American College of Medical Genetics and Genomics (ACMG) and published in the literature (Li et al., 2017; MacArthur et al., 2014; Richards et al., 2008). P or LP variants were routinely reported to couples and variants of uncertain significance (VUS) were provided only if the partner of the VUS carrier also had a P or LP variant in the same gene.

## 3 RESULTS

### 3.1 Population demographics

During the study period, we performed 2,923 ECS in patients seeking ART. The main reasons for their ART requests included chromosome abnormalities, family history of genetic diseases, recurrent spontaneous abortion or infertility, and previous adverse pregnancy outcomes. In total, 2,840 (97.16%) partners from 1,420 couples and 83 (2.84%) individuals underwent testing. Twelve (0.81%) of the 1,485 female patients tested were pregnant at the time of screening. Among these ongoing pregnancies, one had been achieved via PGT‐M and the others had been established through natural conception. It should be noted that although we prepared a complete genetic counseling and prenatal genetic diagnosis plan for pregnant ARCs (as described in the section “Clinical principles and practice”) no pregnant ARC was identified in our study. The mean age of the patients tested was 33.1 years (range: 20–63). Approximately 97.95% (*n* = 2,863) of the patients tested reported their ethnicity as Chinese Han, 0.31% (*n* = 9) as one of the five Chinese ethnic groups (Korean, Zhuang, Zang, Yao and She) and the other 1.74% (*n* = 51) did not report their ethnicity. The average sequencing depth for the samples was >100‐fold, covering more than 96% of the target capture regions with 20 more reads.

### 3.2 Disease carrier frequencies

Among the 2,923 individuals screened, 46.73% (*n* = 1,366) were found to be the carriers of at least one of the 135 conditions. Nearly 10% (*n* = 292) of the tested individuals were the carriers for two of the selected conditions, while 2.8% (*n* = 81) of the tested individuals carried variants associated with more than 3 of the selected conditions (Table 1). The average carrier burden was 0.63 per sample. A previous ECS study in a Chinese population indicated that 27.49% of individuals were positive carriers for at least one disease [29]. The increased positive rate obtained from the current study may be explained by the increased number of diseases included in our screening panel.

**Table 1:** The positive rates of 121 recessive diseases and 14 X‐linked diseases in the 2,923 tested individuals

Positive conditions	Number	Percentage (%)
0	1557	53.27
1	993	33.97
2	292	9.99
3	72	2.46
4	8	0.27
5	1	0.03

Since the majority of tested individuals were Han Chinese, the carrier frequency of selected conditions was only estimated for the 2,836 Han individuals without a family history. The most common disease carried by individuals was the *SLC25A13*(OMIM: 603859, reference sequence: NM_014251.2) related Citrin deficiency, with a carrier rate of 3.91% (*n* = 111). Here, the carrier frequencies of many disorders and genes are reported for the first time in the Han Chinese population, including: *GJB2*(OMIM: 121011, reference sequence: NM_004004.5) (*n* = 107, 3.74%), *SLC22A5*(OMIM: 603377, reference sequence: NM_003060.3) (*n* = 44, 1.54%), *PMM2*(OMIM: 601785, reference sequence: NM_000303.2) (*n* = 34,1.19%) (Table 2, Tables S2 and S3).

**Table 2:** Carrier frequencies of the top 15 diseases in the 2,836 Han Chinese individuals without a family history

Disease	Number	Carrier frequency (%)	1 in_
Citrin deficiency	111	3.91	26
GJB2‐related nonsyndromic hearing loss	106	3.74	27
Krabbe disease	80	2.82	36
Usher syndrome type 2A	76	2.68	38
Alpha‐thalassemia	66	2.33	43
Wilson disease	66	2.33	43
Pendred syndrome	63	2.22	46
Phenylalanine hydroxylase (PAH) deficiency (including PKU)	55	1.94	52
Oculocutaneous albinism, types 1A, 1B, 2, and 4	54	1.90	53
Congenital disorder of glycosylation	52	1.83	55
Systemic primary carnitine deficiency	44	1.55	65
CYP1B1‐related glaucoma	40	1.41	71
Spinal muscular atrophy (SMA)	34	1.20	84
Polycystic kidney disease, autosomal recessive type	32	1.13	89
Usher syndrome type 1	32	1.13	89

### 3.3 Action taken by ARCs after ECS

Overall, 1,462 couples were tested. Among these, 42 couples followed the female first protocol, in which the male was only tested if the female was positive. In the remaining 1,420 couples, 71.5% (*n* = 1,016) were carrier couples, that is, one or both partners were carriers for at least one condition. Surprisingly, 2.26% (*n* = 33) of the tested couples were identified as ARCs and these couples carried genetic markers associated with 19 different diseases. Among the ARCs, 29 couples carried P or LP variants in the same gene in both partners and 4 couples carried an X‐linked P or LP variant in the female partner. Information about the ARCs, including high‐risk diseases and genes, age at the time of ECS, reason for seeking ART, the action taken after ECS, and the length of time since receiving ECS results until the time of the survey (March 2020) are given in Table 3. Among the 33 ARCs, 19 couples reported no family history of the genetic disorders screened by the ECS test and the other 14 had an affected birth with the recessive disorder identical to that identified in the ECS test. All ARCs received genetic counseling and recommendations for PGT‐M. At the time of the survey, 21 (63.6%) ARCs underwent PGT‐M to avoid an affected pregnancy, of which 10 reported no family history of disorders. Four (12.1%) ARCs underwent PGT for aneuploidies (PGT‐A) or for structural chromosome rearrangements (PGT‐SR). It should be noted that ARC 8 reluctantly gave up PGT‐M because they could not find a reference, which is necessary for PGT‐M linkage analysis. However, they planned to obtain prenatal diagnosis for the at‐risk gene after PGT‐SR. One ARC decided to obtain prenatal diagnosis after becoming pregnant naturally. The other seven ARCs (21.2%) had not yet decided which PGT procedure to undergo. Of these, ARC 14 and 23 had not yet made a decision because the cause of their previous adverse pregnancy outcomes remains unclear; the other ARCs did not pursue additional treatment at this center after receiving ECS results.

**Table 3:** Actions taken of ARCs after the ECS test

ID	At‐risk disease	At‐risk gene	Ages at ECS (male/female)	Reason for seeking ART	Original PGT plan	Action taken after ECS	Length of time since receiving ECS results (months)
1	Congenital disorder of glycosylation	*PMM2*	36/32	Adverse childbirth of unknown cause of disease	NA	NA	32
2	Maple syrup urine disease, types Ia, Ib, and II	*DBT*	40/35	Adverse childbirth of monogenic diseases (Maple syrup urine disease)	PGT‐M	Trying spontaneous pregnancy and prenatally diagnosis	32
3	Glucose‐6‐phosphate dehydrogenase deficiency	*G6PD*	40/40	Recurrent miscarriage	PGT‐A	PGT‐A	26
4	Citrullinemia type I	*ASS1*	30/28	Adverse childbirth of monogenic diseases (*ASS1*)	PGT‐M	PGT‐M	25
5	Chronic granulomatous disease, X‐linked	*CYBB*	30/29	Adverse childbirth of monogenic diseases (*CYBB*)	PGT‐M	PGT‐M	21
6	Usher syndrome type 2A	*USH2A*	26/26	Chromosomal abnormalities in female	PGT‐SR	PGT‐M	21
7	Phenylalanine hydroxylase deficiency	*PAH*	27/26	Adverse pregnancy with chromosomal abnormalities	PGT‐A	NA	20
8	Isolated methylmalonic acidemia	*MMUT*	41/41	Adverse childbirth of monogenic diseases (*MMUT*)	PGT‐M	PGT‐M	20
9	Glucose‐6‐phosphate dehydrogenase deficiency	*G6PD*	38/29	Adverse pregnancy with chromosomal abnormalities	PGT‐SR	PGT‐SR + PNDx (No reference for linkage analysis)	19
10	Citrin deficiency	*SLC25A13*	34/31	Recurrent miscarriage	PGT‐A	PGT‐A	19
11	Citrin deficiency	*SLC25A13*	35/30	Recurrent miscarriage and adverse pregnancy with chromosomal abnormalities	PGT‐A	PGT‐M	19
12	Citrin deficiency	*SLC25A13*	31/30	Recurrent miscarriage and chromosomal abnormalities in male	PGT‐SR	PGT‐SR	17
13	Alpha‐thalassemia	*HBA1/HBA2*	33/31	Infertility	NA	PGT‐M	17
14	Fabry disease	*GLA*	28/28	Adverse childbirth of unknown cause of disease	NA	NA	15
15	Citrin deficiency	*SLC25A13*	44/41	Recurrent embryo transplant failure	PGT‐A	PGT‐M	15
16	Phenylalanine hydroxylase deficiency	*PAH*	29/29	Recurrent miscarriage	PGT‐A	PGT‐M	14
17	GJB2‐related nonsyndromic hearing loss, DFNB1A	*GJB2*	27/25	Adverse pregnancy and chromosomal abnormalities in female	PGT‐SR	PGT‐M	13
18	Spinal muscular atrophy (SMA)	*SMN1*	37/36	Adverse childbirth of monogenic diseases (*SMN1*)	PGT‐M	PGT‐M	13
19	Glycogen storage disease type I, subtypes Ia and Ib	*G6PC*	47/41	Adverse childbirth of monogenic diseases (*G6PC*)	PGT‐M	PGT‐M	13
20	Spinal muscular atrophy (SMA)	*SMN1*	31/29	Adverse childbirth of monogenic diseases (*SMN1*)	PGT‐M	NA	13
21	Alpha‐thalassemia	*HBA1/HBA2*	46/35	Recurrent miscarriage	PGT‐A	NA	12
22	Citrin deficiency	*SLC25A13*	32/31	Recurrent miscarriage	PGT‐A	PGT‐M	12
23	Metachromatic leukodystrophy	*ARSA*	36/34	Adverse childbirth of unknown cause of disease	NA	NA	11
24	Autosomal recessive congenital ichthyosis type 1	*TGM1*	27/29	Adverse childbirth of monogenic diseases (*TGM1*)	PGT‐M	PGT‐M	10
25	Spinal muscular atrophy (SMA)	*SMN1*	27/26	Adverse childbirth of monogenic diseases (*SMN1*)	PGT‐M	PGT‐M	10
26	Methylmalonic acidemia with homocystinuria, type cblC	*MMACHC*	31/32	Adverse childbirth of monogenic diseases (*MMACHC*)	PGT‐M	PGT‐M	9
27	Peroxisome biogenesis disorders, Zellweger syndrome spectrum	*PEX1*	26/29	Adverse childbirth of monogenic diseases (*PEX1*)	PGT‐M	NA	9
28	Alpha‐thalassemia	*HBA1/HBA2*	35/34	Adverse childbirth of monogenic diseases (*HBA1*/*HBA2*)	PGT‐M	PGT‐M	8
29	Spinal muscular atrophy (SMA)	*SMN1*	32/28	Adverse childbirth of monogenic diseases (*SMN1*)	PGT‐M	PGT‐M	8
30	Spinal muscular atrophy (SMA)	*SMN1*	35/30	Adverse childbirth of monogenic diseases (*SMN1*)	PGT‐M	PGT‐M	8
31	Citrin deficiency	*SLC25A13*	35/35	Chromosomal abnormalities in female	PGT‐SR	PGT‐M	8
32	Oculocutaneous albinism, types 1A, 1B	*TYR*	29/26	Chromosomal abnormalities in female	PGT‐SR	PGT‐M	8
33	Citrin deficiency	*SLC25A13*	36/34	Recurrent miscarriage	PGT‐A	PGT‐M	7

Note

*PMM2*(OMIM: 601785, reference sequence: NM_000303.2), *DBT* (OMIM: 248610, reference sequence: NM_001918.3), *G6PD*(OMIM: 305900, reference sequence: NM_001042351.2), *ASS1*(OMIM: 603470, reference sequence: NM_000050.4), *CYBB*(OMIM: 300481, reference sequence: NM_000397.3), *USH2A*(OMIM: 608400, reference sequence: NM_206933.2), *PAH*(OMIM: 612349, reference sequence: NM_000277.1), *MMUT*(OMIM: 609058, reference sequence: NM_000255.3), *SLC25A1*(OMIM: 603859, reference sequence: NM_014251.2), *HBA1*(OMIM: 141800, reference sequence: NM_000558.4),*HBA2*(OMIM: 141850, reference sequence: NM_000517.4), *GLA*(OMIM: 300644, reference sequence: NM_000169.2), *GJB2*(OMIM: 121011, reference sequence: NM_004004.5), *SMN1*(OMIM: 600354, reference sequence: NM_000344.3), *G6PC*(OMIM: 613742, reference sequence: NM_000151.3), *ARSA*(OMIM: 607574, reference sequence: NM_000487.5), *TGM1*(OMIM: 190195, reference sequence: NM_000359.2), *MMACHC*(OMIM: 609831, reference sequence: NM_015506.2), *PEX1*(OMIM: 602136, reference sequence: NM_000466.2), *TYR*(OMIM: 606933, reference sequence: NM_000372.4).

Abbreviations: ARC, at‐risk couple; ART, assisted reproductive technology; ECS, expanded carrier screening; PGT, preimplantation genetic testing; PGT‐A, PGT for aneuploidies; PGT‐M, preimplantation genetic testing for monogenetic conditions.

### 3.4 Yield estimation of the ECS panel

According to Guo and Gregg's study in 2019 (Guo & Gregg, 2019), we estimated the yields of screening 187 AR genes in our ECS panel of 2,836 Han Chinese individuals with no family history. The 14 X‐linked recessive genes were excluded from the analysis because of limitations inherent to Guo's model (Guo & Gregg, 2019). The variant carrier rate (VCR) is the proportion of individuals who carry a certain P or LP variant. The top three recurrent variants were c.235del(p.Leu79Cysfs*3) in *GJB2* at a frequency of 1/42, c.2T>C (p.Met1?) in *SLC25A13*at a frequency of 1/43, and c.1901T>C (p.Leu634Ser) in *GALC*(OMIM: 606890, reference sequence: NM_000153.3) at a frequency of 1/45 (Table 4). The VCRs across all 187 AR genes are listed in Table S4.

**Table 4:** Top 15 variants by variant carrier rates (VCR) of the selected genes in the 2,836 Han Chinese individuals without a family history

Gene	Variant location	Variant	Allele count *N*	VCR	1 in _
				%	
*GJB2*	NM_004004.5	c.235del(p.Leu79Cysfs*3)	69	2.40	42
*SLC25A13*	NM_014251.2	c.2T>C(p.Met1?)	66	2.33	43
*GALC*	NM_000153.3	c.1901T>C(p.Leu634Ser)	66	2.26	45
*SMN1*	NM_000344.3	Exon7 heterozygous deletion	39	1.38	73
*HBA1/HBA2*	NM_000558.4/NM_000517.4	Heterozygous α3.7 Deletion	36	1.27	79
*CYP1B1*	NM_000104.3	c.319C>G(p.Leu107Val)	34	1.20	84
*MLC1*	NM_015166.3	c.65G>A(p.Arg22Gln)	29	1.02	98
*SLC26A4*	NM_000441.1	c.919‐2A>G	28	0.99	102
*SLC22A5*	NM_003060.3	c.1400C>G(p.Ser467Cys)	26	0.92	110
*USH2A*	NM_206933.2	c.2802T>G(p.Cys934Trp)	24	0.85	119
*SLC25A13*	NM_014251.2	c.852_855del(p.Met285Profs*2)	22	0.78	129
*COL4A3*	NM_000091.4	c.4793T>G(p.Leu1598Arg)	17	0.60	167
*GJB2*	NM_004004.5	c.299_300del(p.His100Argfs*14)	17	0.60	167
*CAPN3*	NM_000070.2	c.2120A>G(p.Asp707Gly)	16	0.56	178
*HBA1/HBA2*	NM_000558.4/NM_000517.4	Heterozygous SEA Deletion	15	0.53	190

Note

*GJB2*(OMIM: 121011), *SLC25A13*(OMIM: 603859), *GALC*(OMIM: 606890), *SMN1*(OMIM: 600354), *HBA1*(OMIM: 141800), *HBA2*(OMIM: 141850), *CYP1B1*(OMIM: 601771), *MLC1*(OMIM: 605908), *SLC26A4*(OMIM: 605646), *SLC22A5*(OMIM: 603377), *USH2A*(OMIM: 608400), *COL4A3*(OMIM: 120070), *CAPN3*(OMIM: 114240).

Next, the VCRs were used to estimate gene carrier rates (GCRs) for each gene. The GCR value of a gene is the estimated proportion of individuals carrying one or more P or LP variants in that gene. The GCR for each of the 187 AR genes are also listed in Table S5. For illustrative purposes, Table 5 shows the top 15 GCR genes. Unsurprisingly, the *SLC25A13* gene has the highest GCR at 3.87%. In general, the GCRs decline rapidly, with only 17 genes with a GCR >1% (Table 6).

**Table 5:** Top 15 genes by gene carrier rate (GCR) in 2,836 Han Chinese individuals without a family history

*Genes*	GCR (%)	1 in _
*SLC25A13*	3.87	26
*GJB2*	3.66	28
*GALC*	2.74	37
*USH2A*	2.65	38
*ATP7B*	2.30	44
*HBA1/HBA2*	2.20	46
*SLC26A4*	2.14	47
*PAH*	1.92	53
*SMN1*	1.54	65
*SLC22A5*	1.41	71
*CYP1B1*	1.20	84
*TYR*	1.19	84
*PMM2*	1.16	87
*PKHD1*	1.12	90
*GAA*	1.09	92

Note

*SLC25A13*(OMIM: 603859, reference sequence: NM_014251.2), *GJB2*(OMIM: 121011, reference sequence: NM_004004.5), *GALC*(OMIM: 606890, reference sequence: NM_000153.3), *USH2A*(OMIM: 608400, reference sequence: NM_206933.2), *ATP7B*(OMIM: 606882, reference sequence: NM_000053.3), *HBA1*(OMIM: 141800, reference sequence: NM_000558.4), *HBA2*(OMIM: 141850, reference sequence: NM_000517.4), *SLC26A4*(OMIM: 605646, reference sequence: NM_000441.1), *PAH*(OMIM: 612349, reference sequence: NM_000277.1), *SMN1*(OMIM: 600354, reference sequence: NM_000344.3), *SLC22A5*(OMIM: 603377, reference sequence: NM_003060.3), *CYP1B1*(OMIM: 601771, reference sequence: NM_000104.3), *TYR*(OMIM: 606933, reference sequence: NM_000372.4), *PMM2*(OMIM: 601785, reference sequence: NM_000303.2), *PKHD1*(OMIM: 606702, reference sequence: NM_138694.3), *GAA*(OMIM: 606800, reference sequence: NM_000152.3).

**Table 6:** Number of genes with GCR > 1%, GCR > 0.5%, and GCR > 0.1% in 2,836 Han Chinese individuals without a family history. The cumulative carrier rates (CCR) and at‐risk couple rates (ACR) were also calculated and listed below

Gene sets	*N*	CCR (%)	ACR (%)
>1%	17	27.12	0.7346789
>0.5%	31	34.12	0.8102914
>0.1%	111	44.55	0.8565335
All 187 genes	187	45.91	0.8580375

Abbreviation: GCR, gene carrier rate.

**Figure 2:**
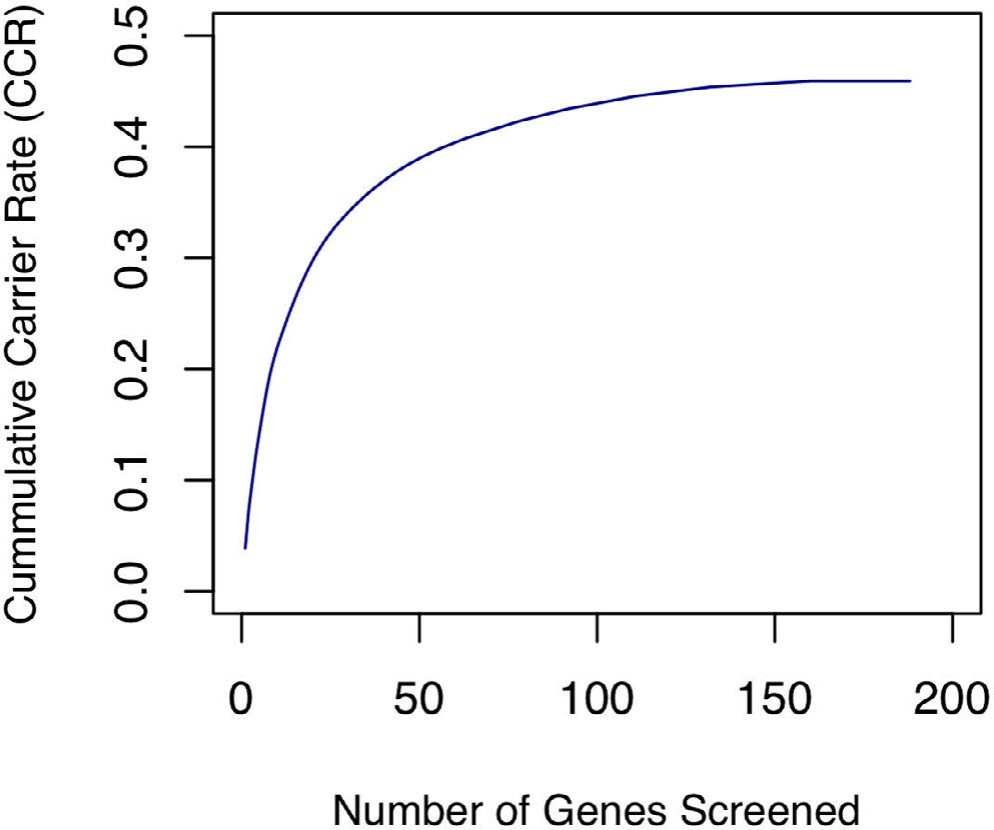
Cumulative carrier rates (CCR) for the selected 187 autosomal recessive genes. Genes are ranked in descending order based on gene carrier rate (GCR) for the 2,836 Han Chinese individuals without a family history

Using these GCRs, cumulative carrier rates (CCRs) for various sets of genes were also calculated. The CCR is an estimation of the detection rate of a hypothetical carrier‐screening panel. The CCR of our 187 AR genes was 45.91% for the study population, which is relatively high compared to the CCRs for the 416 genes selected by Guo and Gregg (2019) (36.5% in East Asian to 65% in Ashkenazi Jewish populations). This implies that our selection of genes is relatively well fit for the Han Chinese population. Furthermore, as shown in Figure 2, as more genes are added to the panel there is an initial rapid increase in the CCR attributed to a small number of genes with high GCRs. This is then followed by a long tail corresponding to genes that contribute asymptotically to the maximum CCR. Roughly, 90% of the CCR can be attributed to the top 68 GCR genes. ACOG recently recommend that genes with a GCR >1% are the preferred genes for use in ECS ("Committee Opinion No. 690 Summary: Carrier Screening in the Age of Genomic Medicine," 2017); however, the results in Table 6 show that this stringent GCR threshold would reduce the CCR by ~41% and therefore may eliminate the diagnoses of many carriers.

Last, at‐risk couple rate (ACR), which is the probability that both members of a couple are carriers for P or LP variants in the same gene, was estimated for gene sets with GCR >1.0%, GCR >0.5%, or GCR >0.1%(Table 6). Similar to previous reports (Guo & Gregg, 2019), the ACR values indicate that the screening for only 31 genes with GCRs >0.5% (81.0 of 10,000 couples) will identify 94.4% of the ARCs that would be identified by screening all 187 genes (85.8 of 10,000 couples).

## 4 DISCUSSION

From our practice, we found that patients who were seeking PGT were more likely to participate in ECS compared with ordinary reproductive couples as well as other members of the infertile population. This may be because most PGT couples have fertility difficulties, which makes them more anxious for a genetically healthy child. Thus, they are more interested in information about their reproductive risks. ECS helps these couples feel autonomous and well prepared for the birth of a child (Kraft, Duenas, Wilfond, & Goddard, 2019). Second, for ARCs identified by ECS, PGT‐M provides an effective way to avoid the identified genetic conditions along with other genetic abnormalities at no additional harm to embryos. Third, the cost of ECS (about $435 per individual) is relatively modest compared with that of IVF and PGT (less than 10%) while the potential benefit is considerable.

The selected panel screens many disorders, increasing the importance of established recommendations already familiar to healthcare providers. Our results showed that genetic counseling, both pre‐and post‐test, informed patients of the available screening options along with their benefits and downsides. Couples were informed that in most cases the ECS test only reports carrier status for mutations that are known to have a well‐defined phenotype, that is, P/LP variants and VUS are not reported unless the partner of the VUS carrier also possesses a P/LP variant in the same gene. Our experiences of integrating ECS into ART indicated that the genetic counseling throughout this process should address the following issues: (a) given the limitation of the linkage‐analysis based technology used in PGT‐M (Gould & Griffin, 2017), it cannot be guaranteed that a couple who tests positive for a condition will be eligible for PGT‐M, for example, if the P/LP variants they carry are de novo or if they are not able to provide a reference. However, the ineligible ARCs can still benefit from ECS through other means such as prenatal diagnosis; (b) patients should be informed of the potential limitations before screening. For instance, there is residual risk associated with NGS of certain genes or their surrounding area and many genes contain pathogenic variants in the intronic region, that is, *SLC26A4*(OMIM: 605646, reference sequence: NM_000441.1) and *ATP7B*(OMIM: 606882, reference sequence: NM_000053.2) (Pera et al., 2008; Todorov, Balakrishnan, Savov, Socha, & Schmidt, 2016); (c) a family history for the identified disorders of the ARCs should be clarified during genetic counseling. This helps couples take action when pathogenic variants with incomplete penetrance are found. Moreover, family history can influence the decision making of ARCs in which one member carries a disease‐causing variant and the other has a VUS found in the same gene; (d) the incidence of chromosomal abnormalities in infertile patients is higher than that in the general population. Due to the technological limitations of NGS, certain chromosomal abnormalities, such as 47, XXX, and mosaics of 45, X, may lead to false negatives when screening for genes on the abnormal chromosomes. Therefore, it is necessary to establish the chromosome status of participants during genetic counseling and an appropriate disclaimer should be added to the ECS report.

Genetic counseling is essential for the proper implementation of ECS (Archibald et al., 2018; Zhao et al., 2019). In this study, genetic counseling was provided by genetic clinicians in the IVF clinic. The interpretation of VUS is a substantial challenge during counseling (Yuan et al., 2009; Zhao et al., 2019). On one hand, informing patients that they carry a VUS may generate unnecessary anxiety, as the majority of VUS are eventually determined to be non‐disease‐causing (Martin et al., 2015; Mastantuoni et al., 2018). On the other hand, VUS cannot be altogether ignored since some of them are pathogenic (Yuan et al., 2009; Zhao et al., 2019). Currently, various IVF centers treat VUS results obtained from ECS differently. A reproductive medicine center in Europe that performs ECS by targeted‐NGS is reporting VUS to patients by default (Abuli et al., 2016). Another center, where VUS is reported if a pathogenic variant in the same gene is found in the partner, details that the option to ask for PGT‐M was sometimes introduced in this context (Martin et al., 2015). Although the ACMG and European Society of Human Genetics (ESHG) both recommend against reporting of VUS in most cases (Green et al., 2013; Henneman et al., 2016), whether these recommendations hold equally true in the context of PGT can be debated. The identification of a pathogenic variant in one member of the couple in combination with a VUS in the other member may become common with ECS. Although traditionally the presence of VUS is not an adequate indication for PGT‐M, the circumstances outlined above warrant further assessment of VUS and subsequent PGT, especially for couples with a family history of genetic disorders. It is our opinion that this is the only case in which VUS should be reported to couples and acted upon, which highlights the importance of ECS as a test for reproductive couples, rather than for individuals. Following the guideline proposed by Martin et al. (2015), we reported VUS to the couples only if the partner of the VUS carrier also had a P or LP variant in the same gene. The genetic counselors then worked to independently evaluate various lines of evidence to reclassify the VUS and explained their findings to the patients. PGT‐M was introduced as an option during counseling if the VUS was reclassified as disease‐causing.

The selection of genes to be included in an ECS panel is usually an issue of hot debate. Until now, no specific decision could be made for a specific region or population (Beauchamp et al., 2019; Bristow et al., 2019; Mastantuoni et al., 2018). Adding genes to an ECS panel allows for more ARCs to be identified; however, it also increases costs substantially due to the detection techniques and downstream interpretation and counseling, as well as increases anxiety in the patient (Guo & Gregg, 2019). Although the technical cost of NGS is becoming increasingly negligible, genes that cannot be detected by NGS with adequate sensitivity require additional means of detection and add extra costs. For instance, *DMD* (OMIM: 300377), *SMN1*, and *HBA* require fragment length confirmation. The greatest expense of ECS may be derived from the cost of variant interpretation and genetic counseling, which deserves earnest consideration. Our results indicate that increases to ACR are minimal when genes of low GCR (<0.5%) are added to the screen. Therefore, an ancestry‐specific ECS panel, which screens for genes that make a large contribution to ACR may adequately balance costs and benefits, especially for clinics whose patients are relatively ethnically homogeneous. Furthermore, as recommended by the previous research (Antonarakis, 2019; Plantinga et al., 2019; Schuurmans et al., 2019), offering couple‐based ECS which reports positive “couple‐results” only may be another promising approach to reduce the costs incurred by variant interpretation and counseling.

## 5 CONCLUSION

In this study, we launched a pilot population‐based ECS for 135 severe recessive Mendelian conditions, the aim of which was to inform the clinical utility of ECS in Chinese ART patients. Our results suggest a need to implement ECS in the ART population, since more than 46% of tested individuals were carriers for at least one selected disease. Furthermore, the ACR for the 187 AR genes in the ECS panel was estimated to be 85.8 out of 10,000 couples. This rate is comparable to the frequency of neonates affected by Down syndrome, screening for which is offered in routine government‐subsidized antenatal tests in China (Hook, Cross, & Schreinemachers, 1983). From a clinical perspective, carrier identification by ECS prompted 10 ARCs to turn to PGT‐M to prevent affected pregnancies, accounting for 47.6% of the ARCs who underwent PGT‐M (21 couples). ARCs who underwent PGT‐A or PGT‐SR instead of PGT‐M could also benefit from post‐test genetic counseling that introduces the option of prenatal diagnosis. Moreover, positive ECS results may increase the opportunities for antenatal intervention as well as optimize newborn and infant outcomes by helping to rapidly diagnose and immediately intervene or begin treatment after birth (Edwards et al., 2015; Mastantuoni et al., 2018). Taken together, our study suggests that ECS holds significant clinical utility for Chinese ART patients. In addition, by estimating the yields of the ECS panel for ART patients of Han ethnicity, we inform the selection of genes that should be included in ESC and provide important implications for the design of ECS panels.

## CONFLICT OF INTEREST

The authors declare no conflict of interest.

## AUTHOR CONTRIBUTIONS

YX performed the data collection, interpreted the data, wrote and revised the manuscript. GC participated in variant interpretation. GC and CL contributed to the data interpretation and manuscript preparation. CL, JW, and YZ contributed to the genetic counseling and the recruitment of the patient. SZ and MX involved in data collection and contributed to the critical revision of the manuscript for important intellectual content. WZ contributed to the recruitment of the patient. YZ and XS designed and supervised the study. All authors critically reviewed the manuscript.

## Supporting information

Table S1

Table S2

Table S3

Table S4

Table S5

## Data Availability

The data that support the findings of this study are available from the corresponding author (XS) upon reasonable request.
