# Supplementary material for: Expanded carrier screening in Chinese patients seeking the help of assisted reproductive technology
Source: Mol Genet Genomic Med. 2020 Jun 23;8(9):e1340. doi: 10.1002/mgg3.1340 (PMC7507411; doi:10.1002/mgg3.1340)
Supplement: Supplementary file 1 — Table S1 [file MGG3-8-e1340-s001.pdf]

Table S1. Recessive diseases/genes included in the ECS test.

Disease Name	OMIM Numbers	Genetic Mode	Genes
Glucose-6-phosphate dehydrogenase (G6PD) deficiency	300908	XLR	G6PD(OMIM:305900)
Bardet-Biedl syndrome types 1, 2 and 10	209900, 615981, 615987	AR	BBS1(OMIM:209901), BBS10(OMIM:610148), BBS2(OMIM:606151)
GJB2-related nonsyndromic hearing loss, DFNB1A	220290	AR	GJB2(OMIM:121011)
Dihydropyrimidine dehydrogenase deficiency	274270	AR	DPYD(OMIM:612779)
Mucolipidosis type IV	252650	AR	MCOLN1(OMIM:605248)
GLB1-related disorders	230500, 230600, 230650, 253010	AR	GLB1(OMIM:611458)
Ornithine transcarbamylase (OTC) deficiency	311250	XLR	OTC(OMIM:300461)
Usher syndrome type 1	276900, 276904, 601067, 602083	AR	MYO7A(OMIM:276903), USH1C(OMIM:605242), CDH23(OMIM:605516), PCDH15(OMIM:605514), USH1G(OMIM:607696)
Usher syndrome type 2A	276901	AR	USH2A(OMIM:608400)
Usher syndrome type 3A	276902, 614504	AR	CLRN1(OMIM:606397)
Dihydrolipoamide dehydrogenase (DLD) deficiency	246900	AR	DLD(OMIM:238331)
Maple syrup urine disease, types Ia, Ib and II	248600	AR	BCKDHA(OMIM:608348), BCKDHB(OMIM:248611), DBT(OMIM:248610)
Glycogen storage disease type I, subtypes Ia and Ib (GSDI, von Gierke disease)	232200, 232220	AR	SLC37A4(OMIM:602671), G6PC(OMIM:613742)
Glycogen storage disease type II (GSDII, Pompe Disease)	232300	AR	GAA(OMIM:606800)
Glycogen storage disease type III (GSDIII, Cori disease)	232400	AR	AGL(OMIM:610860)
Glycogen storage disease type IV	232500	AR	GBE1(OMIM:607839)
Glycogen storage disease type V (GSDV, McArdle disease)	232600	AR	PYGM(OMIM:608455)
Methylmalonic acidemia with homocystinuria, type cblC (cblC)	277400	AR	MMACHC(OMIM:609831)
Phenylalanine hydroxylase (PAH) deficiency (including PKU)	261600	AR	PAH(OMIM:612349)

Hexosaminidase A (HEX A) deficiency (including Tay-Sachs disease)	272800	AR	HEXA(OMIM:606869)
Polycystic kidney disease, autosomal recessive type	263200	AR	PKHD1(OMIM:606702)
Wilson disease	277900	AR	ATP7B(OMIM:606882)
Systemic primary carnitine deficiency	212140	AR	SLC22A5(OMIM:603377)
Very long-chain acyl-CoA dehydrogenase (VLCAD) deficiency	201475	AR	ACADVL(OMIM:609575)
Mucopolysaccharidosis type I (MPSI) (including Hurler syndrome, Hurler-Scheie syndrome,Scheie syndrome)	607014, 607015, 607016	AR	IDUA(OMIM:252800)
Mucopolysaccharidosis type II (MPS II, Hunter syndrome)	309900	XLR	IDS(OMIM:300823)
Mucopolysaccharidosis type III, subtypes IIIA, IIIB, IIIC and IIID (MPS III, Sanfilippo syndrome types A, B, C and D)	252900, 252920, 252930, 252940	AR	SGSH(OMIM:605270), NAGLU(OMIM:609701), HGSNAT(OMIM:610453), GNS(OMIM:607664)
Mucopolysaccharidosis type IV, subtypes IVA and IVB (MPS IV, Morquio syndrome types A and B)	253000, 253010	AR	GALNS(OMIM:612222)
Mucopolysaccharidosis type VI (MPS VI, Maroteaux-Lamy syndrome)	253200	AR	ARSB(OMIM:611542)
Mucopolysaccharidosis type VII (MPS VII, Sly syndrome)	253220	AR	GUSB(OMIM:611499)
Alpha-thalassemia	604131	AR	HBA1(OMIM:141800), HBA2(OMIM:141850)
Pendred syndrome	274600	AR	SLC26A4(OMIM:605646)
POLG-related disorders	203700, 613662, 607459, 258450	AR	POLG(OMIM:174763)
Spinal muscular atrophy (SMA) (including Werdnig-Hoffman disease, Dubowitz disease, Kugelberg-Welander disease)	253300, 271150	AR	SMN1(OMIM:600354)
Neuronal ceroid lipofuscinosis (NCL, Batten disease)	256730, 204500, 609270, 204200, 256731, 601780, 610951, 600143, 610003, 610127	AR	CTSD(OMIM:116840), MFSD8(OMIM:611124), CLN3(OMIM:607042), CLN5(OMIM:608102), CLN6(OMIM:606725), CLN8(OMIM:607837), PPT1(OMIM:600722), TPP1(OMIM:607998)

Congenital disorder of glycosylation	603147, 608799, 606056, 266265, 607091, 603585, 608540, 610768, 212065, 602579, 608093, 614750	AR	ALG6(OMIM:604566), DPM1(OMIM:603503), MOGS(OMIM:601336), SLC35C1(OMIM:605881), B4GALT1(OMIM:137060), SLC35A1(OMIM:605634), ALG1(OMIM:605907), DOLK(OMIM:610746), PMM2(OMIM:601785), MPI(OMIM:154550), DPAGT1(OMIM:191350)
Familial hemophagocytic lymphohistiocytosis	603553, 608898	AR	PRF1(OMIM:170280), UNC13D(OMIM:608897)
Glutaric acidemia type II, types IIA, IIB and IIC (GA2, multiple acyl-CoA dehydrogenase deficiency, MADD)	231680, 231680, 231680	AR	ETFA(OMIM:608053), ETFDH(OMIM:231675), ETFB(OMIM:130410)
Familial hyperinsulinism	256450, 601820	AR	ABCC8(OMIM:600509), KCNJ11(OMIM:600937)
Limb-girdle muscular dystrophy type 2, subtypes 2A, 2C, 2D and 2E (LGMD2)	253600, 253700, 608099, 604286	AR	CAPN3(OMIM:114240), SGCG(OMIM:608896), SGCA(OMIM:600119), SGCB(OMIM:600900)
Niemann-Pick disease types A, B, C1 and C2	257200, 607616, 257220, 607625	AR	SMPD1(OMIM:607608), NPC1(OMIM:607623), NPC2(OMIM:601015)
Propionic acidemia	606054	AR	PCCA(OMIM:232000), PCCB(OMIM:232050)
Short-chain acyl-CoA dehydrogenase deficiency (SCAD deficiency)	201470	AR	ACADS(OMIM:606885)
X-linked agammaglobulinemia (XLA, Bruton's agammaglobulinemia)	300755	XLR	BTK(OMIM:300300)
Ataxia-telangiectasia (AT, Louis-Bar syndrome)	208900	AR	ATM(OMIM:607585)
Biotinidase deficiency	253260	AR	BTD(OMIM:609019)
Carnitine palmitoyltransferase 1A deficiency (CPT1A deficiency)	255120	AR	CPT1A(OMIM:600528)
Carnitine palmitoyltransferase II deficiency (CPT II deficiency)	608836, 600649, 255110	AR	CPT2(OMIM:600650)
Chediak-Higashi syndrome	214500	AR	LYST(OMIM:606897)
Citrin deficiency	605814, 603471	AR	SLC25A13(OMIM:603859)
Citrullinemia type I	215700	AR	ASS1(OMIM:603470)

Cystinosis	219800, 219900, 219750	AR	CTNS(OMIM:606272)
Ehlers-Danlos syndrome, cardiac valvular form (EDS cardiac valvular form)	225320	AR	COL1A2(OMIM:120160)
Epidermolysis bullosa dystrophica, autosomal recessive	226600	AR	COL7A1(OMIM:120120)
Fabry disease	301500	XLR	GLA(OMIM:300644)
Galactosemia	230400	AR	GALT(OMIM:606999)
Glutaric acidemia type I (GA I)	231670	AR	GCDH(OMIM:608801)
Krabbe disease (globoid cell leukodystrophy)	245200	AR	GALC(OMIM:606890)
Medium-chain acyl-CoA dehydrogenase deficiency	201450	AR	ACADM(OMIM:607008)
Arginase deficiency	207800	AR	ARG1(OMIM:608313)
Congenital adrenal hypoplasia, X-linked (X-linked adrenal hypoplasia congenita, X-linked AHC)	300200	XLR	NR0B1(OMIM:300473)
Alpha-1-antitrypsin deficiency (AAT deficiency)	613490	AR	SERPINA1(OMIM:107400)
Ocular albinism, X-linked (XLOA)	300500	XLR	GPR143(OMIM:300808)
Oculocutaneous albinism, types 1A, 1B, 2 and 4 (OCA; OCA1A, OCA1B, OCA2, OCA4)	203100, 606952, 203200, 606574	AR	TYR(OMIM:606933), OCA2(OMIM:611409), SLC45A2(OMIM:606202)
Chronic granulomatous disease, X-linked (X-linked CGD)	306400	XLR	CYBB(OMIM:300481)
Chronic granulomatous disease, autosomal recessive (autosomal recessive CGD)	233700, 233710, 233690	AR	NCF1(OMIM:608512), CYBA(OMIM:608508), NCF2(OMIM:608515)
Congenital myasthenic syndrome (CMS)	254210, 601462, 608930, 605809, 616324, 608931, 603034, 254300, 616326	AR	CHRNE(OMIM:100725), COLQ(OMIM:603033), DOK7(OMIM:610285), RAPSN(OMIM:601592), CHAT(OMIM:118490), CHRNA1(OMIM:100690)
Isolated methylmalonic acidemia (MMA)	251100, 251110, 251000	AR	MMAB(OMIM:607568), MMAA(OMIM:607481), MMUT(OMIM:609058)
Severe congenital neutropenia, autosomal recessive (autosomal recessive SCN, including SCN3 and SCN4)	610738, 612541	AR	G6PC3(OMIM:611045), HAX1(OMIM:605998)

Peroxisome biogenesis disorders, Zellweger syndrome spectrum (PBD, ZSS)	214100, 601539, 614862, 614863	AR	PEX6(OMIM:601498), PEX1(OMIM:602136)
Adenosine deaminase deficiency (ADA deficiency)	102700	AR	ADA(OMIM:608958)
Alpha-mannosidosis	248500	AR	MAN2B1(OMIM:609458)
Argininosuccinate lyase deficiency (ASL deficiency)	207900	AR	ASL(OMIM:608310)
SLC26A2-related disorders (including atelosteogenesis type II, achondrogenesis type IB, diastrophic dysplasia and multiple epiphyseal dysplasia-4)	256050, 600972, 222600, 226900	AR	SLC26A2(OMIM:606718)
Autosomal recessive congenital ichthyosis type 1 (ARCI1)	242300	AR	TGM1(OMIM:190195)
Beta-hemoglobinopathies (including beta-thalassemia and sickle cell disease)	613985, 603903	AR	HBB(OMIM:141900)
Fanconi anemia, complementation group C (FA-C)	227645	AR	FANCC(OMIM:613899)
Homocystinuria	236200	AR	CBS(OMIM:613381)
Hypophosphatasia	241500, 241510	AR	ALPL(OMIM:171760)
Immunodeficiency with hyper-IgM type 1 (HIGM1)	308230	XLR	CD40LG(OMIM:300386)
Isovaleric acidemia	243500	AR	IVD(OMIM:607036)
Long-chain 3-hydroxyacyl-CoA dehydrogenase deficiency	609016	AR	HADHA(OMIM:600890)
X-linked lymphoproliferative syndrome type 1 (XLP1)	308240	XLR	SH2D1A(OMIM:300490)
Metachromatic leukodystroph	250100	AR	ARSA(OMIM:607574)
Mucolipidosis II alpha/beta and Mucolipidosis III alpha/beta	252500, 252600	AR	GNPTAB(OMIM:607840)
Nijmegen breakage syndrome (NBS)	251260	AR	NBN(OMIM:602667)
Pyruvate carboxylase deficiency (PC deficiency)	266150	AR	PC(OMIM:608786)
Rhizomelic chondrodysplasia punctata type 1 (RCDP1)	215100	AR	PEX7(OMIM:601757)
Sandhoff disease	268800	AR	HEXB(OMIM:606873)
Shwachman-Diamond syndrome (SDS)	260400	AR	SBDS(OMIM:607444)
Sjögren-Larsson syndrome	270200	AR	ALDH3A2(OMIM:609523)
Smith-Lemli-Opitz syndrome， SLOS	270400	AR	DHCR7(OMIM:602858)
Tyrosine hydroxylase deficiency (TH deficiency)	605407	AR	TH(OMIM:191290)
Tyrosinemia type I	276700	AR	FAH(OMIM:613871)
WAS-related disorders (including Wiskott-Aldrich syndrome, X-linked congenital neutropenia and X-linked thrombocytopenia)	301000, 300299, 313900	XLR	WAS(OMIM:300392)

Beta-ketothiolase deficiency	203750	AR	ACAT1(OMIM:607809)
Holocarboxylase synthetase deficiency (HLCS deficiency)	253270	AR	HLCS(OMIM:609018)
Laron syndrome	262500	AR	GHR(OMIM:600946)
Lysosomal acid lipase deficiency (LAL deficiency)	278000	AR	LIPA(OMIM:613497)
Alport syndrome, autosomal recessive (autosomal recessive Alport syndrome, ARAS)	203780	AR	COL4A3(OMIM:120070), COL4A4(OMIM:120131)
ATP8B1 deficiency (including progressive familial intrahepatic cholestasis type 1 and benign recurrent intrahepatic cholestasis type 1)	211600, 243300	AR	ATP8B1(OMIM:602397)
ABCB11 deficiency (including progressive familial intrahepatic cholestasis type 2 and benign recurrent intrahepatic cholestasis type 2)	601847, 605479	AR	ABCB11(OMIM:603201)
Congenital dystroglycanopathies (including Fukuyama congenital muscular dystrophy, muscle-eye-brain disease and Walker-Warburg syndrome)	611615, 253800, 613152, 611588, 613154, 608840, 253280 , 613151, 613157 , 617123, 236670, 613155, 609308, 613150, 613156, 613158	AR	FKTN(OMIM:607440), LARGE1(OMIM:603590), POMGNT1(OMIM:606822), POMT1(OMIM:607423), POMT2(OMIM:607439)
Combined pituitary hormone deficiency types 1, 2 and 3	613038, 262600, 221750	AR	POU1F1(OMIM:173110), PROP1(OMIM:601538), LHX3(OMIM:600577)
Joubert syndrome	608629, 610188	AR	AHI1(OMIM:608894), CEP290(OMIM:610142)
Nephrotic syndrome, type 1,2	256300, 600995	AR	NPHS1(OMIM:602716), NPHS2(OMIM:604766)
Glycine encephalopathy	605899	AR	GLDC(OMIM:238300), AMT(OMIM:238310)
Primary hyperoxaluria	259900, 260000	AR	AGXT(OMIM:604285), GRHPR(OMIM:604296)

BH4-deficient hyperphenylalaninemia (BH4-deficient HPA)	261640, 233910, 261630	AR	PTS(OMIM:612719), QDPR(OMIM:612676), GCH1(OMIM:600225)
Abetalipoproteinemia	200100	AR	MTTP(OMIM:157147)
Hereditary motor and sensory neuropathy with agenesis of the corpus callosum (HMSN/ACC, Andermann syndrome)	218000	AR	SLC12A6(OMIM:604878)
Aspartylglucosaminuria (AGU)	208400	AR	AGA(OMIM:613228)
Ataxia with vitamin E deficiency	277460	AR	TTPA(OMIM:600415)
Autosomal recessive congenital ichthyosis type 4 (ARCI4, including subtypes 4A and 4B, harlequin ichthyosis)	601277, 242500	AR	ABCA12(OMIM:607800)
Cerebrotendinous xanthomatosis	213700	AR	CYP27A1(OMIM:606530)
Congenital amegakaryocytic thrombocytopenia	604498	AR	MPL(OMIM:159530)
Cystic fibrosis	219700, 277180	AR	CFTR(OMIM:602421)
3-hydroxy-3-methylglutaryl-CoA lyase deficiency (HMG-CoA lyase deficiency)	246450	AR	HMGCL(OMIM:613898)
Ehlers-Danlos syndrome, type VI	225400	AR	PLOD1(OMIM:153454)
Ethylmalonic encephalopathy	602473	AR	ETHE1(OMIM:608451)
CYP1B1-related glaucoma	231300	AR	CYP1B1(OMIM:601771)
Hereditary fructose intolerance	229600	AR	ALDOB(OMIM:612724)
Hyperornithinemia-hyperammonemia-homocitrullinuria syndrome	238970	AR	SLC25A15(OMIM:603861)
PLA2G6-associated neurodegeneration (PLAN, including infantile neuroaxonal dystrophy 1)	256600, 610217	AR	PLA2G6(OMIM:603604)
Leukoencephalopathy with vanishing white matter	603896	AR	EIF2B5(OMIM:603945)
Lowe syndrome	309000	XLR	OCRL(OMIM:300535)
Lysinuric protein intolerance	222700	AR	SLC7A7(OMIM:603593)
MLC1-related megalencephalic leukoencephalopathy with subcortical cysts	604004	AR	MLC1(OMIM:605908)
Mitochondrial trifunctional protein deficiency	609015	AR	HADHB(OMIM:143450)
Multiple sulfatase deficiency	272200	AR	SUMF1(OMIM:607939)
Nonaka myopathy	605820	AR	GNE(OMIM:603824)
Sialic acid storage disease (including Salla disease)	269920, 604369	AR	SLC17A5(OMIM:604322)
Autosomal recessive spastic ataxia of Charlevoix-Saguenay	270550	AR	SACS(OMIM:604490)
L1 syndrome	307000	XLR	L1CAM(OMIM:308840)
X-linked adrenoleukodystrophy	300100	XLR	ABCD1(OMIM:300371)
