# Supplementary material for: Expanded carrier screening in Chinese patients seeking the help of assisted reproductive technology
Source: Mol Genet Genomic Med. 2020 Jun 23;8(9):e1340. doi: 10.1002/mgg3.1340 (PMC7507411; doi:10.1002/mgg3.1340)
Supplement: Supplementary file 2 — Table S2 [file MGG3-8-e1340-s002.pdf]

Table S2. The carrier frequencies of 135 recessive diseases included in the ECS test in the 2,836 Han ethnicity individuals without family history.

Disease	N	Frequency	1 in _
Citrin deficiency	111	0.03913963	26
GJB2-related nonsyndromic hearing loss, DFNB1A	106	0.03737659	27
Krabbe disease (globoid cell leukodystrophy)	80	0.02820874	36
Usher syndrome type 2A	76	0.02679831	38
Wilson disease	66	0.02327221	43
Alpha-thalassemia	66	0.02327221	43
Pendred syndrome	63	0.02221439	46
Phenylalanine hydroxylase (PAH) deficiency (including PKU)	55	0.01939351	52
Oculocutaneous albinism, types 1A, 1B, 2 and 4 (OCA; OCA1A, OCA1B, OCA2, OCA4)	54	0.0190409	53
Congenital disorder of glycosylation	52	0.01833568	55
Systemic primary carnitine deficiency	44	0.01551481	65
CYP1B1-related glaucoma	40	0.01410437	71
Spinal muscular atrophy (SMA) (including Werdnig-Hoffman disease, Dubowitz disease, Kugelberg-Welander disease)	34	0.01198872	84
Polycystic kidney disease, autosomal recessive type	32	0.0112835	89
Usher syndrome type 1	32	0.0112835	89
Glycogen storage disease type II (GSDII, Pompe Disease)	31	0.01093089	92
Glutaric acidemia type II, types IIA, IIB and IIC (GA2, multiple acyl-CoA dehydrogenase deficiency, MADD)	30	0.01057828	95
MLC1-related megalencephalic leukoencephalopathy with subcortical cysts	30	0.01057828	95
Methylmalonic acidemia with homocystinuria, type cblC (cblC)	28	0.00987306	102
Familial hemophagocytic lymphohistiocytosis	27	0.00952045	106
Nonaka myopathy	26	0.00916784	110
Joubert syndrome	26	0.00916784	110
Isolated methylmalonic acidemia (MMA)	25	0.00881523	114
Glycogen storage disease type I, subtypes Ia and Ib (GSDI, von Gierke disease)	24	0.00846262	119
Cerebrotendinous xanthomatosis	24	0.00846262	119
Cystic fibrosis	23	0.00811001	124
Limb-girdle muscular dystrophy type 2, subtypes 2A, 2C, 2D and 2E (LGMD2)	23	0.00811001	124
Alport syndrome, autosomal recessive (autosomal recessive Alport syndrome, ARAS)	21	0.0074048	136
Niemann-Pick disease types A, B, C1 and C2	20	0.00705219	142
Hypophosphatasia	18	0.00634697	158
Congenital dystroglycanopathies (including Fukuyama congenital muscular dystrophy, muscle-eye-brain disease and Walker-Warburg syndrome)	18	0.00634697	158
BH4-deficient hyperphenylalaninemia (BH4-deficient HPA)	15	0.00528914	190
Beta-hemoglobinopathies (including beta-thalassemia and sickle cell disease)	15	0.00528914	190
Congenital myasthenic syndrome (CMS)	15	0.00528914	190
Peroxisome biogenesis disorders, Zellweger syndrome spectrum (PBD, ZSS)	15	0.00528914	190
Epidermolysis bullosa dystrophica, autosomal recessive	15	0.00528914	190
Nephrotic syndrome, type 1,2	14	0.00493653	203
Mucopolysaccharidosis type III, subtypes IIIA, IIIB, IIIC and IIID (MPS III, Sanfilippo syndrome types A, B, C and D)	13	0.00458392	219
Primary hyperoxaluria	13	0.00458392	219
Bardet-Biedl syndrome types 1, 2 and 10	13	0.00458392	219
Glutaric acidemia type I (GA I)	12	0.00423131	237
Dihydropyrimidine dehydrogenase deficiency	12	0.00423131	237
Very long-chain acyl-CoA dehydrogenase (VLCAD) deficiency	11	0.0038787	258
Short-chain acyl-CoA dehydrogenase deficiency (SCAD deficiency)	11	0.0038787	258

Hereditary fructose intolerance	10	0.00352609	284
Propionic acidemia	10	0.00352609	284
Medium-chain acyl-CoA dehydrogenase deficiency	10	0.00352609	284
Galactosemia	9	0.00317348	316
Mucolipidosis II alpha/beta and Mucolipidosis III alpha/beta	9	0.00317348	316
Neuronal ceroid lipofuscinosis (NCL, Batten disease)	8	0.00282087	355
Biotinidase deficiency	8	0.00282087	355
Sjögren-Larsson syndrome	7	0.00246827	406
Tyrosine hydroxylase deficiency (TH deficiency)	7	0.00246827	406
Smith-Lemli-Opitz syndrome， SLOS	7	0.00246827	406
Maple syrup urine disease, types Ia, Ib and II	7	0.00246827	406
Metachromatic leukodystroph	7	0.00246827	406
Shwachman-Diamond syndrome (SDS)	6	0.00211566	473
Holocarboxylase synthetase deficiency (HLCS deficiency)	5	0.00176305	568
Mucopolysaccharidosis type I (MPSI)<br>(including Hurler syndrome, Hurler-Scheie syndrome,Scheie syndrome)	5	0.00176305	568
Carnitine palmitoyltransferase II deficiency (CPT II deficiency)	5	0.00176305	568
Isovaleric acidemia	5	0.00176305	568
Chronic granulomatous disease, autosomal recessive (autosomal recessive CGD)	5	0.00176305	568
PLA2G6-associated neurodegeneration (PLAN, including infantile neuroaxonal dystrophy 1)	5	0.00176305	568
Glycogen storage disease type V (GSDV, McArdle disease)	5	0.00176305	568
Alpha-1-antitrypsin deficiency (AAT deficiency)	5	0.00176305	568
Sandhoff disease	5	0.00176305	568
Glycogen storage disease type III (GSDIII, Cori disease)	5	0.00176305	568
Autosomal recessive congenital ichthyosis type 1 (ARCI1)	5	0.00176305	568
Hexosaminidase A (HEX A) deficiency (including Tay-Sachs disease)	4	0.00141044	709
GLB1-related disorders	4	0.00141044	709
Fanconi anemia, complementation group C (FA-C)	4	0.00141044	709
Congenital amegakaryocytic thrombocytopenia	4	0.00141044	709
Mucopolysaccharidosis type VI (MPS VI, Maroteaux-Lamy syndrome)	4	0.00141044	709
Lysosomal acid lipase deficiency (LAL deficiency)	4	0.00141044	709
Ehlers-Danlos syndrome, type VI	4	0.00141044	709
Citrullinemia type I	3	0.00105783	946
Autosomal recessive spastic ataxia of Charlevoix-Saguenay	3	0.00105783	946
Familial hyperinsulinism	3	0.00105783	946
Adenosine deaminase deficiency (ADA deficiency)	3	0.00105783	946
Ethylmalonic encephalopathy	3	0.00105783	946
Glucose-6-phosphate dehydrogenase (G6PD) deficiency	3	0.00105783	946
Mitochondrial trifunctional protein deficiency	3	0.00105783	946
Severe congenital neutropenia, autosomal recessive (autosomal recessive SCN, including SCN3 and SCN4)	3	0.00105783	946
Mucopolysaccharidosis type VII (MPS VII, Sly syndrome)	3	0.00105783	946
Cystinosis	3	0.00105783	946
Homocystinuria	3	0.00105783	946
Beta-ketothiolase deficiency	3	0.00105783	946
Chediak-Higashi syndrome	3	0.00105783	946
Hereditary motor and sensory neuropathy with agenesis of the corpus callosum (HMSN/ACC, Andermann syndrome)	3	0.00105783	946
ABCB11 deficiency (including progressive familial intrahepatic cholestasis type 2 and benign recurrent intrahepatic cholestasis type 2)	2	0.00070522	1418
Alpha-mannosidosis	2	0.00070522	1418
Abetalipoproteinemia	2	0.00070522	1418
Glycine encephalopathy	2	0.00070522	1418

Mucopolysaccharidosis type IV, subtypes IVA and IVB (MPS IV, Morquio syndrome types A and B)	2	0.00070522	1418
Nijmegen breakage syndrome (NBS)	2	0.00070522	1418
POLG-related disorders	2	0.00070522	1418
Leukoencephalopathy with vanishing white matter	2	0.00070522	1418
Lysinuric protein intolerance	2	0.00070522	1418
Pyruvate carboxylase deficiency (PC deficiency)	2	0.00070522	1418
3-hydroxy-3-methylglutaryl-CoA lyase deficiency (HMG-CoA lyase deficiency)	1	0.00035261	2836
Dihydrolipoamide dehydrogenase (DLD) deficiency	1	0.00035261	2836
Ataxia with vitamin E deficiency	1	0.00035261	2836
ATP8B1 deficiency (including progressive familial intrahepatic cholestasis type 1 and benign recurrent intrahepatic cholestasis type 1)	1	0.00035261	2836
Autosomal recessive congenital ichthyosis type 4 (ARCI4, including subtypes 4A and 4B, harlequin ichthyosis)	1	0.00035261	2836
Fabry disease	1	0.00035261	2836
Ataxia-telangiectasia (AT, Louis-Bar syndrome)	1	0.00035261	2836
Aspartylglucosaminuria (AGU)	1	0.00035261	2836
Mucolipidosis type IV	1	0.00035261	2836
Combined pituitary hormone deficiency types 1, 2 and 3	1	0.00035261	2836
Tyrosinemia type I	1	0.00035261	2836
SLC26A2-related disorders (including atelosteogenesis type II, achondrogenesis type IB, diastrophic dysplasia and multiple epiphyseal dysplasia-4)	1	0.00035261	2836
Hyperornithinemia-hyperammonemia-homocitrullinuria syndrome	1	0.00035261	2836
Glycogen storage disease type IV	1	0.00035261	2836
Argininosuccinate lyase deficiency (ASL deficiency)	1	0.00035261	2836
Long-chain 3-hydroxyacyl-CoA dehydrogenase deficiency	1	0.00035261	2836
Arginase deficiency	0	0	Inf
Usher syndrome type 3A	0	0	Inf
X-linked agammaglobulinemia (XLA, Bruton’s agammaglobulinemia)	0	0	Inf
Lowe syndrome	0	0	Inf
Ocular albinism, X-linked (XLOA)	0	0	Inf
Laron syndrome	0	0	Inf
Carnitine palmitoyltransferase 1A deficiency (CPT1A deficiency)	0	0	Inf
Immunodeficiency with hyper-IgM type 1 (HIGM1)	0	0	Inf
Ehlers-Danlos syndrome, cardiac valvular form (EDS cardiac valvular form)	0	0	Inf
Sialic acid storage disease (including Salla disease)	0	0	Inf
X-linked lymphoproliferative syndrome type 1 (XLP1)	0	0	Inf
L1 syndrome	0	0	Inf
Ornithine transcarbamylase (OTC) deficiency	0	0	Inf
WAS-related disorders (including Wiskott-Aldrich syndrome, X-linked congenital neutropenia and X-linked thrombocytopenia)	0	0	Inf
Congenital adrenal hypoplasia, X-linked (X-linked adrenal hypoplasia congenita, X-linked AHC)	0	0	Inf
Rhizomelic chondrodysplasia punctata type 1 (RCDP1)	0	0	Inf
Mucopolysaccharidosis type II (MPS II, Hunter syndrome)	0	0	Inf
Chronic granulomatous disease, X-linked (X-linked CGD)	0	0	Inf
X-linked adrenoleukodystrophy	0	0	Inf
Multiple sulfatase deficiency	0	0	Inf
