# Supplementary material for: Expanded carrier screening in Chinese patients seeking the help of assisted reproductive technology
Source: Mol Genet Genomic Med. 2020 Jun 23;8(9):e1340. doi: 10.1002/mgg3.1340 (PMC7507411; doi:10.1002/mgg3.1340)
Supplement: Supplementary file 3 — Table S3 [file MGG3-8-e1340-s003.pdf]

Table S3. The carrier frequencies of 201 genes included in the ECS test in the 2,836 Han ethnicity individuals without family history

Gene	N	Frequency	1 in _
SLC25A13	111	0.039139633	26
GJB2	106	0.037376587	27
GALC	80	0.028208745	36
USH2A	76	0.026798307	38
ATP7B	66	0.023272214	43
HBA1|HBA2	66	0.023272214	43
SLC26A4	63	0.022214386	46
PAH	55	0.019393512	52
SLC22A5	44	0.01551481	65
CYP1B1	40	0.014104372	71
PMM2	34	0.011988717	84
TYR	34	0.011988717	84
SMN1	34	0.011988717	84
PKHD1	32	0.011283498	89
GAA	31	0.010930889	92
MLC1	30	0.010578279	95
MMACHC	28	0.009873061	102
ETFDH	27	0.009520451	106
GNE	26	0.009167842	110
CYP27A1	24	0.008462623	119
CFTR	23	0.008110014	124
CAPN3	21	0.007404795	136
MMUT	21	0.007404795	136
G6PC	19	0.006699577	150
COL4A3	18	0.006346968	158
ALPL	18	0.006346968	158
UNC13D	16	0.005641749	178
HBB	15	0.00528914	190
COL7A1	15	0.00528914	190
CEP290	15	0.00528914	190
CDH23	14	0.00493653	203
PTS	14	0.00493653	203
OCA2	13	0.004583921	219
PEX1	13	0.004583921	219
SMPD1	13	0.004583921	219
GCDH	12	0.004231312	237
DPYD	12	0.004231312	237
ACADVL	11	0.003878702	258
PRF1	11	0.003878702	258
ACADS	11	0.003878702	258
AHI1	11	0.003878702	258
ACADM	10	0.003526093	284
ALDOB	10	0.003526093	284
MYO7A	10	0.003526093	284
GNPTAB	9	0.003173484	316
GALT	9	0.003173484	316
BBS2	9	0.003173484	316
NPHS1	8	0.002820874	355

AGXT	8	0.002820874	355
BTD	8	0.002820874	355
TH	8	0.002820874	355
SLC45A2	7	0.002468265	406
DHCR7	7	0.002468265	406
ALDH3A2	7	0.002468265	406
SGSH	7	0.002468265	406
ARSA	7	0.002468265	406
PCCB	6	0.002115656	473
NPHS2	6	0.002115656	473
NPC1	6	0.002115656	473
MOGS	6	0.002115656	473
PCDH15	6	0.002115656	473
POMT1	6	0.002115656	473
SBDS	6	0.002115656	473
TGM1	5	0.001763047	568
GRHPR	5	0.001763047	568
AGL	5	0.001763047	568
CPT2	5	0.001763047	568
PLA2G6	5	0.001763047	568
PYGM	5	0.001763047	568
IVD	5	0.001763047	568
HEXB	5	0.001763047	568
IDUA	5	0.001763047	568
HLCS	5	0.001763047	568
POMGNT1	5	0.001763047	568
SERPINA1	5	0.001763047	568
SLC37A4	5	0.001763047	568
RAPSN	5	0.001763047	568
PCCA	4	0.001410437	709
DOK7	4	0.001410437	709
LIPA	4	0.001410437	709
FANCC	4	0.001410437	709
MMAA	4	0.001410437	709
SLC35A1	4	0.001410437	709
BCKDHB	4	0.001410437	709
POMT2	4	0.001410437	709
CHAT	4	0.001410437	709
GLB1	4	0.001410437	709
PLOD1	4	0.001410437	709
MPL	4	0.001410437	709
ARSB	4	0.001410437	709
HEXA	4	0.001410437	709
CLN5	3	0.001057828	946
SLC12A6	3	0.001057828	946
SACS	3	0.001057828	946
ASS1	3	0.001057828	946
G6PD	3	0.001057828	946
COL4A4	3	0.001057828	946
HGSNAT	3	0.001057828	946
FKTN	3	0.001057828	946

ACAT1	3	0.001057828	946
BBS10	3	0.001057828	946
LYST	3	0.001057828	946
DPM1	3	0.001057828	946
ADA	3	0.001057828	946
GUSB	3	0.001057828	946
HAX1	3	0.001057828	946
HADHB	3	0.001057828	946
CYBA	3	0.001057828	946
CTNS	3	0.001057828	946
ABCC8	3	0.001057828	946
CBS	3	0.001057828	946
MAN2B1	2	0.000705219	1418
PC	2	0.000705219	1418
SLC7A7	2	0.000705219	1418
ALG1	2	0.000705219	1418
ALG6	2	0.000705219	1418
ETHE1	2	0.000705219	1418
PEX6	2	0.000705219	1418
ABCB11	2	0.000705219	1418
NCF2	2	0.000705219	1418
POLG	2	0.000705219	1418
NBN	2	0.000705219	1418
ETFA	2	0.000705219	1418
NAGLU	2	0.000705219	1418
EIF2B5	2	0.000705219	1418
MTTP	2	0.000705219	1418
GBE1	2	0.000705219	1418
USH1C	2	0.000705219	1418
MFSD8	2	0.000705219	1418
TPP1	2	0.000705219	1418
GALNS	2	0.000705219	1418
BCKDHA	2	0.000705219	1418
AGA	1	0.000352609	2836
SLC25A15	1	0.000352609	2836
CLN6	1	0.000352609	2836
HMGCL	1	0.000352609	2836
HADHA	1	0.000352609	2836
ATP8B1	1	0.000352609	2836
TTPA	1	0.000352609	2836
DBT	1	0.000352609	2836
COLQ	1	0.000352609	2836
GLDC	1	0.000352609	2836
NPC2	1	0.000352609	2836
DLD	1	0.000352609	2836
SLC26A2	1	0.000352609	2836
ABCA12	1	0.000352609	2836
MPI	1	0.000352609	2836
GLA	1	0.000352609	2836
ATM	1	0.000352609	2836
SGCG	1	0.000352609	2836

PROP1	1	0.000352609	2836
SGCA	1	0.000352609	2836
ASL	1	0.000352609	2836
ETFB	1	0.000352609	2836
QDPR	1	0.000352609	2836
BBS1	1	0.000352609	2836
GNS	1	0.000352609	2836
FAH	1	0.000352609	2836
CHRNE	1	0.000352609	2836
MCOLN1	1	0.000352609	2836
AMT	1	0.000352609	2836
DOLK	0	0	Inf
L1CAM	0	0	Inf
LHX3	0	0	Inf
GHR	0	0	Inf
PPT1	0	0	Inf
NR0B1	0	0	Inf
ABCD1	0	0	Inf
CLN8	0	0	Inf
SH2D1A	0	0	Inf
CTSD	0	0	Inf
MMAB	0	0	Inf
G6PC3	0	0	Inf
LARGE1	0	0	Inf
SUMF1	0	0	Inf
B4GALT1	0	0	Inf
PEX7	0	0	Inf
ARG1	0	0	Inf
BTK	0	0	Inf
NCF1	0	0	Inf
CD40LG	0	0	Inf
GCH1	0	0	Inf
IDS	0	0	Inf
GPR143	0	0	Inf
SGCB	0	0	Inf
CLN3	0	0	Inf
SLC35C1	0	0	Inf
CHRNA1	0	0	Inf
WAS	0	0	Inf
SLC17A5	0	0	Inf
COL1A2	0	0	Inf
OCRL	0	0	Inf
OTC	0	0	Inf
DPAGT1	0	0	Inf
USH1G	0	0	Inf
CLRN1	0	0	Inf
CPT1A	0	0	Inf
KCNJ11	0	0	Inf
CYBB	0	0	Inf
POU1F1	0	0	Inf
