# Supplementary material for: Expanded carrier screening in Chinese patients seeking the help of assisted reproductive technology
Source: Mol Genet Genomic Med. 2020 Jun 23;8(9):e1340. doi: 10.1002/mgg3.1340 (PMC7507411; doi:10.1002/mgg3.1340)
Supplement: Supplementary file 4 — Table S4 [file MGG3-8-e1340-s004.pdf]

Table S4. The variant carrier rate (VCRs) estimated from the data of 2,836 Han Chinese individuals with no family history across the 187 AR genes covered by the ECS test.

gene_symbol	HGVS	VCR	1 in _
GJB2	NM_004004.5:c.235del(p.Leu79Cysfs*3)	0.023977433	41.70588235
SLC25A13	NM_014251.2:c.2T>C(p.Met1?)	0.023272214	42.96969697
GALC	NM_000153.3:c.1901T>C(p.Leu634Ser)	0.022566996	44.3125
HBA1/HBA2	Heterozygous α3.7 Deletion	0.013751763	72.71794872
CYP1B1	NM_000104.3:c.319C>G(p.Leu107Val)	0.012693935	78.77777778
SMN1	exon 7 heterozygous deletion	0.011988717	83.41176471
MLC1	NM_015166.3:c.65G>A(p.Arg22Gln)	0.01022567	97.79310345
SLC26A4	NM_000441.1:c.919-2A>G	0.009873061	101.2857143
SLC22A5	NM_003060.3:c.1400C>G(p.Ser467Cys)	0.009167842	109.0769231
USH2A	NM_206933.2:c.2802T>G(p.Cys934Trp)	0.008462623	118.1666667
SLC25A13	NM_014251.2:c.852_855del(p.Met285Profs*2)	0.007757405	128.9090909
COL4A3	NM_000091.4:c.4793T>G(p.Leu1598Arg)	0.005994358	166.8235294
GJB2	NM_004004.5:c.299_300del(p.His100Argfs*14)	0.005994358	166.8235294
CAPN3	NM_000070.2:c.2120A>G(p.Asp707Gly)	0.005641749	177.25
HBA1/HBA2	Heterozygous SEA Deletion	0.00528914	189.0666667
PMM2	NM_000303.2:c.634A>G(p.Met212Val)	0.00493653	202.5714286
ETFDH	NM_004453.3:c.770A>G(p.Tyr257Cys)	0.004583921	218.1538462
ATP7B	NM_000053.3:c.2975C>T(p.Pro992Leu)	0.004583921	218.1538462
ATP7B	NM_000053.3:c.3316G>A(p.Val1106Ile)	0.004583921	218.1538462
ATP7B	NM_000053.3:c.2333G>T(p.Arg778Leu)	0.004583921	218.1538462
GNE	NM_001128227.2:c.620A>T(p.Asp207Val)	0.004583921	218.1538462
USH2A	NM_206933.2:c.9259G>A(p.Val3087Ile)	0.004231312	236.3333333
SLC26A4	NM_000441.1:c.2168A>G(p.His723Arg)	0.004231312	236.3333333
G6PC	NM_000151.3:c.648G>T(p.Leu216=)	0.004231312	236.3333333
CDH23	NM_022124.5:c.4762C>T(p.Arg1588Trp)	0.003878702	257.8181818
MMACHC	NM_015506.2:c.609G>A(p.Trp203*)	0.003878702	257.8181818
UNC13D	NM_199242.2:c.2588G>A(p.Gly863Asp)	0.003878702	257.8181818
PKHD1	NM_138694.3:c.2507T>C(p.Val836Ala)	0.003173484	315.1111111
HBB	NM_000518.4:c.52A>T(p.Lys18*)	0.002820874	354.5
GALT	NM_000155.3:c.821-7A>G	0.002820874	354.5
USH2A	NM_206933.2:c.8559-2A>G	0.002468265	405.1428571
TYR	NM_000372.4:c.896G>A(p.Arg299His)	0.002468265	405.1428571
SMPD1	NM_000543.4:c.995C>G(p.Pro332Arg)	0.002468265	405.1428571
GNE	NM_001128227.2:c.18T>A(p.Tyr6*)	0.002468265	405.1428571
PAH	NM_000277.1:c.158G>A(p.Arg53His)	0.002115656	472.6666667
SLC25A13	NM_014251.2:c.615+5G>A	0.002115656	472.6666667
PMM2	NM_000303.2:c.430T>C(p.Phe144Leu)	0.002115656	472.6666667
PTS	NM_000317.2:c.259C>T(p.Pro87Ser)	0.002115656	472.6666667
CYP27A1	NM_000784.3:c.410G>A(p.Arg137Gln)	0.002115656	472.6666667
SLC22A5	NM_003060.3:c.51C>G(p.Phe17Leu)	0.002115656	472.6666667
DPYD	NM_000110.3:c.1155_1156del(p.Cys385*)	0.002115656	472.6666667
ALDH3A2	NM_000382.2:c.1157A>G(p.Asn386Ser)	0.001763047	567.2
ACADM	NM_000016.5:c.449_452del(p.Thr150Argfs*4)	0.001763047	567.2
PTS	NM_000317.2:c.286G>A(p.Asp96Asn)	0.001763047	567.2
ALDOB	NM_000035.3:c.1013C>T(p.Ala338Val)	0.001763047	567.2
HBA1/HBA2	Heterozygous α4.2 Deletion	0.001763047	567.2
GALC	NM_000153.3:c.1912G>A(p.Gly638Ser)	0.001763047	567.2
GJB2	NM_004004.5:c.176_191del(p.Gly59Alafs*18)	0.001763047	567.2
TGM1	NM_000359.2:c.420A>G(p.Ile140Met)	0.001410437	709
USH2A	NM_206933.2:c.2653C>T(p.His885Tyr)	0.001410437	709
PAH	NM_000277.2:c.611A>G(p.Tyr204Cys)	0.001410437	709
PAH	NM_000277.2:c.728G>A(p.Arg243Gln)	0.001410437	709

PAH	NM_000277.1:c.721C>T(p.Arg241Cys)	0.001410437	709
PAH	NM_000277.1:c.611A>G(p.Tyr204Cys)	0.001410437	709
SLC25A13	NM_014251.2:c.1177+1G>A	0.001410437	709
ETFDH	NM_004453.3:c.1657T>C(p.Tyr553His)	0.001410437	709
CFTR	NM_000492.3:c.2909G>A(p.Gly970Asp)	0.001410437	709
TYR	NM_000372.4:c.929dup(p.Arg311Lysfs*7)	0.001410437	709
ATP7B	NM_000053.3:c.3443T>C(p.Ile1148Thr)	0.001410437	709
SLC26A4	NM_000441.1:c.697G>C(p.Val233Leu)	0.001410437	709
CYP27A1	NM_000784.3:c.435G>T(p.Gly145=)	0.001410437	709
MMACHC	NM_015506.2:c.80A>G(p.Gln27Arg)	0.001410437	709
MMACHC	NM_015506.2:c.482G>A(p.Arg161Gln)	0.001410437	709
MMUT	NM_000255.3:c.1663G>A(p.Ala555Thr)	0.001410437	709
MMUT	NM_000255.3:c.1106G>A(p.Arg369His)	0.001410437	709
BTD	NM_000060.4:c.44+2T>C	0.001410437	709
DPYD	NM_000110.3:c.220C>T(p.Arg74*)	0.001410437	709
TH	NM_199292.2:c.698G>A(p.Arg233His)	0.001410437	709
GAA	NM_000152.3:c.503G>A(p.Arg168Gln)	0.001410437	709
ALPL	NM_000478.5:c.529G>A(p.Ala177Thr)	0.001410437	709
USH2A	NM_206933.2:c.538T>C(p.Ser180Pro)	0.001057828	945.3333333
HBB	NM_000518.4:c.126_129del(p.Phe42Leufs*19)	0.001057828	945.3333333
CPT2	NM_000098.2:c.1891C>T(p.Arg631Cys)	0.001057828	945.3333333
ETFDH	NM_004453.3:c.250G>A(p.Ala84Thr)	0.001057828	945.3333333
SLC45A2	NM_016180.4:c.1519G>C(p.Val507Leu)	0.001057828	945.3333333
PRF1	NM_001083116.2:c.503G>A(p.Ser168Asn)	0.001057828	945.3333333
OCA2	NM_000275.2:c.1327G>A(p.Val443Ile)	0.001057828	945.3333333
OCA2	NM_000275.2:c.247C>T(p.Gln83*)	0.001057828	945.3333333
COL7A1	NM_000094.3:c.8569G>T(p.Glu2857*)	0.001057828	945.3333333
PKHD1	NM_138694.3:c.6091del(p.Ala2031Leufs*2)	0.001057828	945.3333333
TYR	NM_000372.4:c.1265G>A(p.Arg422Gln)	0.001057828	945.3333333
TYR	NM_000372.4:c.164G>A(p.Cys55Tyr)	0.001057828	945.3333333
ACADS	NM_000017.3:c.1031A>G(p.Glu344Gly)	0.001057828	945.3333333
ATP7B	NM_000053.3:c.3451C>T(p.Arg1151Cys)	0.001057828	945.3333333
SLC35A1	NM_006416.4:c.1A>G(p.Met1?)	0.001057828	945.3333333
NPHS1	NM_004646.3:c.928G>A(p.Asp310Asn)	0.001057828	945.3333333
MOGS	NM_006302.2:c.1212_1239dup(p.Asp414Leufs*17)	0.001057828	945.3333333
CYP27A1	NM_000784.3:c.1263+1G>A	0.001057828	945.3333333
SLC22A5	NM_003060.3:c.760C>T(p.Arg254*)	0.001057828	945.3333333
AHI1	NM_017651.4:c.533_534del(p.Glu178Glyfs*3)	0.001057828	945.3333333
CYP1B1	NM_000104.3:c.1169G>A(p.Arg390His)	0.001057828	945.3333333
MMUT	NM_000255.3:c.1280G>A(p.Gly427Asp)	0.001057828	945.3333333
GCDH	NM_000159.3:c.1064G>A(p.Arg355His)	0.001057828	945.3333333
GJB2	NM_004004.5:c.235delC(p.Leu79Cysfs*3)	0.001057828	945.3333333
GJB2	NM_004004.5:c.508_511dup(p.Ala171Glufs*40)	0.001057828	945.3333333
SBDS	NM_016038.2:c.183_184delinsCT(p.Lys62*)	0.001057828	945.3333333
HBA2	NM_000517.4:c.369C>G(p.His123Gln)	0.001057828	945.3333333
BBS2	NM_031885.3:c.1398-2A>G	0.001057828	945.3333333
RAPSN	NM_005055.4:c.149_153delins24(p.Val50Glufs*114)	0.001057828	945.3333333
ALPL	NM_000478.5:c.979T>C(p.Phe327Leu)	0.001057828	945.3333333
ALPL	NM_000478.4:c.529G>A(p.Ala177Thr)	0.001057828	945.3333333
GRHPR	NM_012203.1:c.83+1G>C	0.000705219	1418
GRHPR	NM_012203.1:c.864_865del(p.Val289Aspfs*22)	0.000705219	1418
ACADVL	NM_000018.3:c.1349G>A(p.Arg450His)	0.000705219	1418
USH2A	NM_206933.2:c.6485+5G>A	0.000705219	1418
USH2A	NM_206933.2:c.8232G>C(p.Trp2744Cys)	0.000705219	1418
USH2A	NM_206933.2:c.11156G>A(p.Arg3719His)	0.000705219	1418

USH2A	NM_206933.2:c.5953G>A(p.Glu1985Lys)	0.000705219	1418
PCCB	NM_000532.4:c.838dup(p.Leu280Profs*11)	0.000705219	1418
HBB	NM_000518.4:c.341T>A(p.Val114Glu)	0.000705219	1418
PAH	NM_000277.2:c.442-1G>A	0.000705219	1418
PAH	NM_000277.1:c.1238G>C(p.Arg413Pro)	0.000705219	1418
PAH	NM_000277.2:c.208_210del(p.Ser70del)	0.000705219	1418
PAH	NM_000277.2:c.721C>T(p.Arg241Cys)	0.000705219	1418
PAH	NM_000277.2:c.1123C>G(p.Gln375Glu)	0.000705219	1418
PAH	NM_000277.1:c.527G>A(p.Arg176Gln)	0.000705219	1418
SLC25A13	NM_014251.2:c.1399C>T(p.Arg467*)	0.000705219	1418
SLC25A13	NM_014251.2:c.1638_1660dup(p.Ala554Glyfs*17)	0.000705219	1418
GNPTAB	NM_024312.4:c.1090C>T(p.Arg364*)	0.000705219	1418
GNPTAB	NM_024312.4:c.99del(p.Ala34Profs*49)	0.000705219	1418
GNPTAB	NM_024312.4:c.1284+1G>T	0.000705219	1418
CAPN3	NM_000070.2:c.802-9G>A	0.000705219	1418
NPHS2	NM_014625.3:c.467dup(p.Leu156Phefs*11)	0.000705219	1418
SLC45A2	NM_016180.4:c.478G>C(p.Asp160His)	0.000705219	1418
PRF1	NM_001083116.2:c.65del(p.Pro22Argfs*29)	0.000705219	1418
PRF1	NM_001083116.1:c.503G>A(p.Ser168Asn)	0.000705219	1418
COL7A1	NM_000094.3:c.3625_3635del(p.Ser1209Leufs*6)	0.000705219	1418
CEP290	NM_025114.3:c.6012-2A>G	0.000705219	1418
MMAA	NM_172250.2:c.650T>A(p.Leu217*)	0.000705219	1418
PKHD1	NM_138694.3:c.8863C>T(p.Arg2955*)	0.000705219	1418
PKHD1	NM_138694.3:c.11740C>T(p.Arg3914*)	0.000705219	1418
PKHD1	NM_138694.3:c.2264C>T(p.Pro755Leu)	0.000705219	1418
CFTR	NM_000492.3:c.1865G>A(p.Gly622Asp)	0.000705219	1418
CFTR	NM_000492.3:c.2907A>C(p.=)	0.000705219	1418
CFTR	NM_000492.3:c.263T>G(p.Leu88*)	0.000705219	1418
CFTR	NM_000492.3:c.293A>G(p.Gln98Arg)	0.000705219	1418
TYR	NM_000372.4:c.229C>G(p.Arg77Gly)	0.000705219	1418
ACADS	NM_000017.3:c.164C>T(p.Pro55Leu)	0.000705219	1418
ALG1	NM_019109.4:c.863-2A>G	0.000705219	1418
PMM2	NM_000303.2:c.448-1_448del	0.000705219	1418
PMM2	NM_000303.2:c.395T>C(p.Ile132Thr)	0.000705219	1418
ATP7B	NM_000053.3:c.2621C>T(p.Ala874Val)	0.000705219	1418
ATP7B	NM_000053.3:c.3517G>A(p.Glu1173Lys)	0.000705219	1418
ATP7B	NM_000053.3:c.2755C>G(p.Arg919Gly)	0.000705219	1418
PTS	NM_000317.2:c.73C>T(p.Arg25*)	0.000705219	1418
SLC26A4	NM_000441.1:c.589G>A(p.Gly197Arg)	0.000705219	1418
SLC26A4	NM_000441.1:c.1226G>A(p.Arg409His)	0.000705219	1418
SLC26A4	NM_000441.1:c.1645dup(p.Arg549Lysfs*15)	0.000705219	1418
NPHS1	NM_004646.3:c.3325C>T(p.Arg1109*)	0.000705219	1418
NPHS1	NM_004646.3:c.3213del(p.Leu1072Phefs*71)	0.000705219	1418
IVD	NM_002225.3:c.1208A>G(p.Tyr403Cys)	0.000705219	1418
IVD	NM_002225.3:c.1183C>T(p.Arg395Cys)	0.000705219	1418
G6PC	NM_000151.3:c.238T>A(p.Phe80Ile)	0.000705219	1418
CYP27A1	NM_000784.3:c.1420C>T(p.Arg474Trp)	0.000705219	1418
CYP27A1	NM_000784.3:c.1214G>A(p.Arg405Gln)	0.000705219	1418
SLC22A5	NM_003060.3:c.1195C>T(p.Arg399Trp)	0.000705219	1418
SLC22A5	NM_003060.3:c.497+1G>T	0.000705219	1418
SLC22A5	NM_003060.3:c.338G>A(p.Cys113Tyr)	0.000705219	1418
MMACHC	NM_015506.2:c.658_660del(p.Lys220del)	0.000705219	1418
PCDH15	NM_033056.3:c.3475dup(p.Met1159Asnfs*12)	0.000705219	1418
AHI1	NM_017651.4:c.1799_1802del(p.Lys600Thrfs*5)	0.000705219	1418
AHI1	NM_017651.4:c.1185_1186del(p.Asn396Cysfs*13)	0.000705219	1418

SMPD1	NM_000543.4:c.1458T>G(p.Ser486Arg)	0.000705219	1418
ACAT1	NM_000019.3:c.1160T>C(p.Ile387Thr)	0.000705219	1418
PEX1	NM_000466.2:c.2391_2392del(p.Arg798Serfs*35)	0.000705219	1418
GALC	NM_000153.3:c.1592G>A(p.Arg531His)	0.000705219	1418
AGXT	NM_000030.2:c.33dup(p.Lys12Glnfs*156)	0.000705219	1418
ADA	NM_000022.2:c.646G>A(p.Gly216Arg)	0.000705219	1418
SGSH	NM_000199.3:c.398del(p.Pro133Argfs*131)	0.000705219	1418
SGSH	NM_000199.3:c.1063G>A(p.Glu355Lys)	0.000705219	1418
HAX1	NM_006118.3:c.430dup(p.Val144Glyfs*5)	0.000705219	1418
GNE	NM_001128227.2:c.1985C>T(p.Ala662Val)	0.000705219	1418
GNE	NM_001128227.2:c.395G>A(p.Arg132His)	0.000705219	1418
SERPINA1	NM_000295.4:c.187C>T(p.Arg63Cys)	0.000705219	1418
SERPINA1	NM_000295.4:c.227_229del(p.Phe76del)	0.000705219	1418
HADHB	NM_000183.2:c.209C>G(p.Ser70*)	0.000705219	1418
GJB2	NM_004004.5:c.232G>A(p.Ala78Thr)	0.000705219	1418
GJB2	NM_004004.5:c.257C>G(p.Thr86Arg)	0.000705219	1418
MTTP	NM_000253.3:c.1868-2A>G	0.000705219	1418
GAA	NM_000152.3:c.2662G>T(p.Glu888*)	0.000705219	1418
GAA	NM_000152.4:c.752_761delinsTGCTGCCCTT(p.Ser251_Ser254delinsLeuLeuProLeu)	0.000705219	1418
GAA	NM_000152.4:c.2024_2026del(p.Asn675del)	0.000705219	1418
GAA	NM_000152.3:c.752_761delinsTGCTGCCCTT(p.Ser251_Ser254delinsLeuLeuProLeu)	0.000705219	1418
GALNS	NM_000512.4:c.1019G>A(p.Gly340Asp)	0.000705219	1418
CYBA	NM_000101.3:c.7C>T(p.Gln3*)	0.000705219	1418
ARSA	NM_000487.5:c.883G>A(p.Gly295Ser)	0.000705219	1418
CBS	NM_000071.2:c.374G>A(p.Arg125Gln)	0.000705219	1418
MYO7A	NM_000260.3:c.2476G>A(p.Ala826Thr)	0.000705219	1418
ALPL	NM_000478.5:c.407G>A(p.Arg136His)	0.000705219	1418
ALPL	NM_000478.4:c.407G>A(p.Arg136His)	0.000705219	1418
TGM1	NM_000359.2:c.607C>T(p.Gln203*)	0.000352609	2836
GRHPR	NM_012203.1:c.598+1G>T	0.000352609	2836
ACADVL	NM_000018.3:c.1837C>T(p.Arg613Trp)	0.000352609	2836
ACADVL	NM_000018.3:c.1059_1060del(p.Gly354Hisfs*4)	0.000352609	2836
ACADVL	NM_000018.3:c.105_109dup(p.Arg37Leufs*26)	0.000352609	2836
ACADVL	NM_000018.3:c.888del(p.Glu297Argfs*56)	0.000352609	2836
ACADVL	NM_000018.3:c.736del(p.Ser246Alafs*10)	0.000352609	2836
ACADVL	NM_000018.3:c.1843C>T(p.Arg615*)	0.000352609	2836
ACADVL	NM_000018.3(ACADVL):c.1349G>A(p.Arg450His)	0.000352609	2836
ACADVL	NM_000018.3:c.1141_1143del(p.Glu381del)	0.000352609	2836
ACADVL	NM_000018.3:c.1748C>T(p.Ser583Leu)	0.000352609	2836
USH2A	NM_206933.2:c.4117del(p.Leu1373Serfs*59)	0.000352609	2836
USH2A	NM_206933.2:c.10859T>C(p.Ile3620Thr)	0.000352609	2836
USH2A	NM_206933.2:c.7594+2del	0.000352609	2836
USH2A	NM_206933.2:c.14287G>A(p.Gly4763Arg)	0.000352609	2836
USH2A	NM_206933.2:c.9469C>T(p.Gln3157*)	0.000352609	2836
USH2A	NM_206933.2:c.12575G>A(p.Arg4192His)	0.000352609	2836
USH2A	NM_206933.2:c.11533C>T(p.Gln3845*)	0.000352609	2836
USH2A	NM_206933.2:c.4576G>A(p.Gly1526Arg)	0.000352609	2836
USH2A	NM_206933.2:c.4251+1G>A	0.000352609	2836
USH2A	NM_206933.2:c.5581G>A(p.Gly1861Ser)	0.000352609	2836
USH2A	NM_206933.2:c.15520-1G>A	0.000352609	2836
USH2A	NM_206933.2:c.3862G>T(p.Glu1288*)	0.000352609	2836
USH2A	NM_206933.2:c.6325+1G>A	0.000352609	2836
USH2A	NM_206933.2:c.13040_13062delinsTCAGAAGTCA(p.Thr4347Ilefs*22)	0.000352609	2836

USH2A	NM_206933.2:c.5191_5192del(p.Met1731Valfs*8)	0.000352609	2836
USH2A	NM_206933.2:c.9145del(p.Val3049Leufs*9)	0.000352609	2836
USH2A	NM_206933.2:c.13822C>T(p.Arg4608*)	0.000352609	2836
USH2A	NM_206933.2:c.3811+2T>G	0.000352609	2836
MAN2B1	NM_000528.3:c.1026+1G>A	0.000352609	2836
MAN2B1	NM_000528.3:c.1048dup(p.His350Profs*26)	0.000352609	2836
PCCB	NM_000532.4:c.493C>T(p.Arg165Trp)	0.000352609	2836
PCCB	NM_000532.4:c.1087T>C(p.Ser363Pro)	0.000352609	2836
PCCB	NM_000532.4:c.1304A>G(p.Tyr435Cys)	0.000352609	2836
PCCB	NM_000532.4:c.1091-2A>G	0.000352609	2836
PCCA	NM_000282.3:c.937C>T(p.Arg313*)	0.000352609	2836
PCCA	NM_000282.3:c.734C>G(p.Ser245*)	0.000352609	2836
PCCA	NM_000282.3:c.1426C>T(p.Arg476*)	0.000352609	2836
PCCA	NM_000282.3:c.2002G>A(p.Gly668Arg)	0.000352609	2836
AGA	NM_000027.3:c.192del(p.Cys64Trpfs*10)	0.000352609	2836
HBB	NM_000518.4:c.-43C>T	0.000352609	2836
HBB	NM_000518.4:c.85dup(p.Leu29Profs*16)	0.000352609	2836
USH1C	NM_005709.3:c.463C>T(p.Arg155*)	0.000352609	2836
USH1C	NM_005709.3:c.586C>T(p.Arg196*)	0.000352609	2836
AGL	NM_000642.2:c.2681+1G>A	0.000352609	2836
AGL	NM_000642.2:c.2950-2del	0.000352609	2836
AGL	NM_000642.2:c.2925_2926dup(p.Ser976Phefs*21)	0.000352609	2836
AGL	NM_000642.2:c.4234del(p.Gly1413Alafs*2)	0.000352609	2836
AGL	NM_000642.2:c.1311dup(p.Asp438Argfs*14)	0.000352609	2836
BBS10	NM_024685.3:c.1391C>G(p.Ser464*)	0.000352609	2836
BBS10	NM_024685.3:c.891_897delinsTTTGT(p.Met298Leufs*5)	0.000352609	2836
BBS10	NM_024685.3:c.1385del(p.Asn462Metfs*26)	0.000352609	2836
PAH	NM_000277.1:c.1222C>T(p.Arg408Trp)	0.000352609	2836
PAH	NM_000277.2:c.1197A>T(p.Val399=)	0.000352609	2836
PAH	NM_000277.1:c.728G>A(p.Arg243Gln)	0.000352609	2836
PAH	NM_000277.1:c.516G>T(p.Gln172His)	0.000352609	2836
PAH	NM_000277.3:c.1223G>A(p.Arg408Gln)	0.000352609	2836
PAH	NM_000277.2:c.1045T>G(p.Ser349Ala)	0.000352609	2836
PAH	NM_000277.1:c.722G>A(p.Arg241His)	0.000352609	2836
PAH	NM_000277.2:c.462C>A(p.Tyr154*)	0.000352609	2836
PAH	NM_000277.2:c.320A>G(p.His107Arg)	0.000352609	2836
PAH	NM_000277.2:c.526C>T(p.Arg176*)	0.000352609	2836
PAH	NM_000277.2:c.975C>G(p.Tyr325*)	0.000352609	2836
PAH	NM_000277.1:c.510T>A(p.His170Gln)	0.000352609	2836
PAH	NM_000277.2:c.1256A>G(p.Gln419Arg)	0.000352609	2836
PAH	NM_000277.2:c.1223G>A(p.Arg408Gln)	0.000352609	2836
PAH	NM_000277.2:c.331C>T(p.Arg111*)	0.000352609	2836
PAH	NM_000277.2:c.1199+1G>C	0.000352609	2836
PAH	NM_000277.2:c.764T>C(p.Leu255Ser)	0.000352609	2836
PAH	NM_000277.1:c.442-1G>A	0.000352609	2836
PAH	NM_000277.2:c.755G>A(p.Arg252Gln)	0.000352609	2836
PAH	NM_000277.1:c.1199G>A(p.Arg400Lys)	0.000352609	2836
PAH	NM_000277.2:c.770G>T(p.Gly257Val)	0.000352609	2836
SLC25A15	NM_014252.3:c.535C>T(p.Arg179*)	0.000352609	2836
SLC25A13	NM_014251.2:c.475C>T(p.Gln159*)	0.000352609	2836
SLC25A13	NM_014251.2:c.1048G>A(p.Asp350Asn)	0.000352609	2836
SLC25A13	NM_014251.2:c.1A>C(p.Met1?)	0.000352609	2836
SLC25A13	NM_014251.2:c.615+1G>A	0.000352609	2836
SLC25A13	NM_014251.2:c.2T>C	0.000352609	2836
SLC25A13	NM_014251.2:c.1108_1109del(p.Met370Valfs*2)	0.000352609	2836

SLC25A13	NM_014251.2:c.1078C>T(p.Arg360*)	0.000352609	2836
SLC25A13	NM014251.2:c.2T>C(p.Met1?)	0.000352609	2836
SLC25A13	NM_014251.2(SLC25A13):c.550C>T(p.Arg184*)	0.000352609	2836
NPC2	NM_006432.3:c.190+5G>A	0.000352609	2836
CPT2	NM_dup000098.2:c.627_630(p.Pro211Valfs*24)	0.000352609	2836
CPT2	NM_000098.2:c.1148T>A(p.Phe383Tyr)	0.000352609	2836
PLA2G6	NM_003560.3:c.1325dup(p.Pro443Thrfs*71)	0.000352609	2836
PLA2G6	NM_003560.2:c.1A>G(p.1Met?)	0.000352609	2836
PLA2G6	NM_003560.2:c.376del(p.Ala126Leufs*4)	0.000352609	2836
PLA2G6	NM_003560.2:c.1771C>T(p.Arg591Trp)	0.000352609	2836
PLA2G6	NM_003560.3:c.1771C>T(p.Arg591Trp)	0.000352609	2836
GNPTAB	NM_024312.4:c.2715+1G>A	0.000352609	2836
GNPTAB	NM_024312.4:c.3094del(p.Thr1032Hisfs*11)	0.000352609	2836
GNPTAB	NM_024312.4:c.2550_2554del(p.Lys850Asnfs*10)	0.000352609	2836
CLN5	NM_006493.2:c.51delG(p.Gln18Lysfs*32)	0.000352609	2836
CLN5	NM_006493.2:c.334C>T(p.Arg112Cys)	0.000352609	2836
CLN5	NM_006493.2:c.128del(p.Gly43Glufs*7)	0.000352609	2836
SLC12A6	NM_133647.1:c.2037C>G(p.Tyr679*)	0.000352609	2836
SLC12A6	NM_133647.1:c.2641C>T(p.Arg881*)	0.000352609	2836
SLC12A6	NM_133647.1:c.61_62del(p.Lys21Aspfs*3)	0.000352609	2836
CAPN3	NM_000070.2:c.1507_1511del(p.Gly503Argfs*72)	0.000352609	2836
CAPN3	NM_000070.2:c.2092C>T(p.Arg698Cys)	0.000352609	2836
CAPN3	NM_000070.2:c.1076C>T(p.Pro359Leu)	0.000352609	2836
NPHS2	NM_014625.3:c.868G>A(p.Val290Met)	0.000352609	2836
NPHS2	NM_014625.3:c.890C>T(p.Ala297Val)	0.000352609	2836
NPHS2	NM_014625.3:c.851C>T(p.Ala284Val)	0.000352609	2836
NPHS2	NM_014625.3:c.211C>T(p.Arg71*)	0.000352609	2836
PYGM	NM_005609.3:c.2393G>A(p.Trp798*)	0.000352609	2836
PYGM	NM_005609.3:c.1363G>C(p.Gly455Arg)	0.000352609	2836
PYGM	NM_005609.2:c.1969+2T>A	0.000352609	2836
PYGM	NM_005609.3:c.2056G>A(p.Gly686Arg)	0.000352609	2836
PYGM	NM_005609.3:c.1094C>T(p.Ala365Val)	0.000352609	2836
DOK7	NM_173660.4:c.539G>C(p.Gly180Ala)	0.000352609	2836
DOK7	NM_173660.4:c.1378dup(p.Gln460Profs*59)	0.000352609	2836
DOK7	NM_173660.4:c.1296_1311del(p.Asp433Argfs*18)	0.000352609	2836
DOK7	NM_173660.4:c.601C>T(p.Arg201*)	0.000352609	2836
LIPA	NM_000235.3:c.1024G>A(p.Gly342Arg)	0.000352609	2836
LIPA	NM_000235.3:c.731_732del(p.Gly244Aspfs*24)	0.000352609	2836
LIPA	NM_000235.3:c.796G>T(p.Gly266*)	0.000352609	2836
LIPA	NM_000235.3:c.966+1G>T	0.000352609	2836
EIF2B5	NM_003907.2:c.196_198delinsTT(p.Val66Phefs*10)	0.000352609	2836
EIF2B5	NM_003907.2:c.1228C>T(p.Gln410*)	0.000352609	2836
HMGCL	NM_000191.2:c.122G>A(p.Arg41Gln)	0.000352609	2836
CDH23	NM_022124.5:c.6085C>T(p.Arg2029Trp)	0.000352609	2836
CDH23	NM_022124.5:c.6049G>A(p.Gly2017Ser)	0.000352609	2836
CDH23	NM_022124.5:c.7610dup(p.Asp2537Glufs*2)	0.000352609	2836
HADHA	NM_000182.4:c.307_314+4delinsTTAG(p.Asp103Leufs*4)	0.000352609	2836
ETFDH	NM_004453.3:c.1691-3C>G	0.000352609	2836
ETFDH	NM_004453.3:c.998A>G(p.Tyr333Cys)	0.000352609	2836
ETFDH	NM_004453.3:c.389A>T(p.Asp130Val)	0.000352609	2836
ETFDH	NM_004453.3:c.1395T>G(p.Tyr465*)	0.000352609	2836
ETFDH	NM_004453.3:c.1167_1179del(p.Gly390Phefs*11)	0.000352609	2836
ETFDH	NM_004453.2:c.1227A>C(p.Leu409Phe)	0.000352609	2836
ETFDH	NM_004453.3:c.1227A>C(p.Leu409Phe)	0.000352609	2836
SLC45A2	NM_016180.4:c.152_153del(p.Val51Glyfs*82)	0.000352609	2836

SLC45A2	NM_016180.4(SLC45A2):c.938dup(p.Met313Ilefs*41)	0.000352609	2836
PRF1	NM_001083116.1:c.228_229del(p.Cys76*)	0.000352609	2836
PRF1	NM_001083116.1:c.1349C>T(p.Thr450Met)	0.000352609	2836
PRF1	NM_001083116.1:c.916G>A(p.Gly306Ser)	0.000352609	2836
PRF1	NM_001083116.1:c.10C>T(p.Arg4Cys)	0.000352609	2836
OCA2	NM_000275.2:c.695dup(p.Ala233Glyfs*26)	0.000352609	2836
OCA2	NM_000275.2:c.1255C>T(p.Arg419Trp)	0.000352609	2836
OCA2	NM_000275.2:c.2359G>A(p.Ala787Thr)	0.000352609	2836
OCA2	NM_000275.2:c.1182+1G>A	0.000352609	2836
OCA2	NM_000275.2:c.2T>C(p.Met1?)	0.000352609	2836
OCA2	NM_000275.2:c.1363A>G(p.Arg455Gly)	0.000352609	2836
OCA2	NM_000275.2:c.701_716del(p.Gly234Valfs*16)	0.000352609	2836
DHCR7	NM_001360.2:c.1210C>T(p.Arg404Cys)	0.000352609	2836
DHCR7	NM_001360.2:c.907G>A(p.Gly303Arg)	0.000352609	2836
DHCR7	NM_001360.2:c.724C>T(p.Arg242Cys)	0.000352609	2836
DHCR7	NM_001360.2:c.1127del(p.Lys376Argfs*37)	0.000352609	2836
DHCR7	NM_001360.2:c.1214del(p.His405Profs*8)	0.000352609	2836
DHCR7	NM_001360.2:c.841G>A(p.Val281Met)	0.000352609	2836
DHCR7	NM_001360.2:c.1112G>A(p.Trp371*)	0.000352609	2836
COL7A1	NM_000094.3:c.4401+2T>A	0.000352609	2836
COL7A1	NM_000094.3:c.6899A>G(p.Gln2300Arg)	0.000352609	2836
COL7A1	NM_000094.3:c.7723G>C(p.Gly2575Arg)	0.000352609	2836
COL7A1	NM_000094.3:c.7411C>T(p.Arg2471*)	0.000352609	2836
COL7A1	NM_000094.3:c.1268del(p.Pro423Argfs*44)	0.000352609	2836
COL7A1	NM_000094.3:c.2292del(p.Ala765Profs*5)	0.000352609	2836
COL7A1	NM_000094.3:c.7474C>T(p.Arg2492*)	0.000352609	2836
COL7A1	NM_000094.3:c.4613G>A(p.Arg1538His)	0.000352609	2836
COL7A1	NM_000094.3:c.8053C>T(p.Arg2685*)	0.000352609	2836
COL7A1	NM_000094.3:c.7023+2T>C	0.000352609	2836
CEP290	NM_025114.3:c.5611_5614del(p.Gln1871Valfs*2)	0.000352609	2836
CEP290	NM_025114.3:c.6400_6403dup(p.Gly2135Aspfs*9)	0.000352609	2836
CEP290	NM_025114.3:c.3574-1G>A	0.000352609	2836
CEP290	NM_025114.3:c.7220_7223del(p.Lys2407Serfs*2)	0.000352609	2836
CEP290	NM_025114.3:c.1078C>T(p.Arg360*)	0.000352609	2836
CEP290	NM_025114.3:c.7328_7332dup(p.Val2445Argfs*3)	0.000352609	2836
CEP290	NM_025114.3:c.1063C>T(p.Gln355*)	0.000352609	2836
CEP290	NM_025114.3:c.6013del(p.Ala2005Profs*11)	0.000352609	2836
CEP290	NM_025114.3:c.2954del(p.Met985Serfs*19)	0.000352609	2836
CEP290	NM_025114.3:c.689_691delinsTT(p.Glu230Valfs*10)	0.000352609	2836
CEP290	NM_025114.3:c.6103_6104del(p.Gln2035Valfs*8)	0.000352609	2836
CEP290	NM_025114.3:c.5798_5799dupAG(p.Gly1934Argfs*7)	0.000352609	2836
CEP290	NM_025114.3:c.6640A>T(p.Lys2214*)	0.000352609	2836
ATP8B1	NM_005603.4:c.2866del(p.Tyr956Thrfs*52)	0.000352609	2836
MMAA	NM_172250.2:c.456del(p.Gly153Valfs*8)	0.000352609	2836
MMAA	NM_172250.2:c.503del(p.Thr168Metfs*10)	0.000352609	2836
PKHD1	NM_138694.3:c.1693+2T>C	0.000352609	2836
PKHD1	NM_138694.3:c.7075dup(p.Ser2359Phefs*20)	0.000352609	2836
PKHD1	NM_138694.3:c.6332+1dup	0.000352609	2836
PKHD1	NM_138694.3:c.11314C>T(p.Arg3772*)	0.000352609	2836
PKHD1	NM_138694.3:c.4321del(p.Ile1441Phefs*19)	0.000352609	2836
PKHD1	NM_138694.3:c.11785+1G>T	0.000352609	2836
PKHD1	NM_138694.3:c.2216C>T(p.Pro739Leu)	0.000352609	2836
PKHD1	NM_138694.3:c.9169del(p.Ala3057Profs*11)	0.000352609	2836
PKHD1	NM_138694.3:c.3062del(p.Leu1021Argfs*29)	0.000352609	2836
PKHD1	NM_138694.3:c.9455del(p.Asn3152Thrfs*10)	0.000352609	2836

PKHD1	NM_138694.3:c.2341C>T(p.Arg781*)	0.000352609	2836
PKHD1	NM_138694.3:c.11295del(p.Leu3767Trpfs*8)	0.000352609	2836
PKHD1	NM_138694.3:c.10036T>C(p.Cys3346Arg)	0.000352609	2836
PKHD1	NM_138694.3:c.2914A>T(p.Lys972*)	0.000352609	2836
CFTR	NM_000492.3:c.3963+1G>C	0.000352609	2836
CFTR	NM_000492.3:c.2907A>C(p.Ala969=)	0.000352609	2836
CFTR	NM_000492.3:c.3988_3989del(p.Gln1330Valfs*6)	0.000352609	2836
CFTR	NM_000492.3:c.377G>A(p.Gly126Asp)	0.000352609	2836
CFTR	NM_000492.3:c.1000C>T(p.Arg334Trp)	0.000352609	2836
CFTR	NM_000492.3:c.2036G>A(p.Trp679*)	0.000352609	2836
CFTR	NM_000492.3:c.223C>T(p.Arg75*)	0.000352609	2836
CFTR	NM_000492.3:c.3209G>A(p.Arg1070Gln)	0.000352609	2836
CFTR	NM_000492.3:c.2901_2908+6del(p.Lys968Trpfs*4)	0.000352609	2836
CFTR	NM_000492.3:c.2249C>T(p.Pro750Leu)	0.000352609	2836
CFTR	NM_000492.3:c.1657C>T(p.Arg553*)	0.000352609	2836
TYR	NM_000372.4:c.230_232dup(p.Arg77_Glu78insGly)	0.000352609	2836
TYR	NM_000372.4:c.832C>T(p.Arg278*)	0.000352609	2836
TYR	NM_000372.4:c.1147G>A(p.Asp383Asn)	0.000352609	2836
TYR	NM_000372.4:c.996G>A(p.Met332Ile)	0.000352609	2836
TYR	NM_000372.4:c.230G>A(p.Arg77Gln)	0.000352609	2836
TYR	NM_000372.4:c.649C>T(p.Arg217Trp)	0.000352609	2836
TYR	NM_000372.4:c.929dupC(p.Arg311Lysfs*7)	0.000352609	2836
TYR	NM_000372.4:c.1352del(p.Tyr451Phefs*34)	0.000352609	2836
TYR	NM_000372.4:c.895C>A(p.Arg299Ser)	0.000352609	2836
TYR	NM_000372.4:c.1204C>T(p.Arg402*)	0.000352609	2836
TYR	NM_000372.4:c.654G>A(p.Trp218*)	0.000352609	2836
TYR	NM_000372.4:c.155G>T(p.Arg52Ile)	0.000352609	2836
TYR	NM_000372.4:c.455C>A(p.Pro152His)	0.000352609	2836
TYR	NM_000372.4:c.1037-7T>A	0.000352609	2836
TYR	NM_000372.4:c.346C>T(p.Arg116*)	0.000352609	2836
ACADS	NM_000017.3:c.1030del(p.Glu344Argfs*30)	0.000352609	2836
ACADS	NM_000017.3:c.904delG(p.Ala302Argfs*26)	0.000352609	2836
ACADS	NM_000017.3:c.46+2T>A	0.000352609	2836
ACADS	NM_000017.3:c.1130C>T(p.Pro377Leu)	0.000352609	2836
ACADS	NM_000017.3:c.164C>T(p.Pro55Leu)	0.000352609	2836
ACADS	NM_000017.3:c.417G>A(p.Trp139*)	0.000352609	2836
SACS	NM_014363.5:c.7088del(p.Leu2363*)	0.000352609	2836
SACS	NM_014363.5:c.6488delT(p.Met2163Serfs*2)	0.000352609	2836
SACS	NM_014363.5:c.10848G>A(p.Trp3616*)	0.000352609	2836
ALDH3A2	NM_000382.2:c.1084del(p.Tyr362Metfs*13)	0.000352609	2836
ALDH3A2	NM_000382.2:c.683G>A(p.Arg228His)	0.000352609	2836
SLC7A7	NM_001126106.2:c.713C>T(p.Ser238Phe)	0.000352609	2836
SLC7A7	NM_001126106.2:c.625+1G>A	0.000352609	2836
ALG6	NM_013339.3:c.723del(p.Phe242Serfs*19)	0.000352609	2836
ALG6	NM_013339.3:c.57G>A(p.Trp19*)	0.000352609	2836
COLQ	NM_005677.3:c.25_26del(p.Leu9Glyfs*124)	0.000352609	2836
PMM2	NM_000303.2:c.484C>T(p.Arg162Trp)	0.000352609	2836
PMM2	NM_000303.2:c.458_462del(p.Ile153Thrfs*27)	0.000352609	2836
PMM2	NM_000303.2:c.634A>G(p.Met212Val)	0.000352609	2836
PMM2	NM_000303.2:c.580C>T(p.Arg194*)	0.000352609	2836
PMM2	NM_000303.2:c.448-2A>T	0.000352609	2836
PMM2	NM_000303.2:c.368G>A(p.Arg123Gln)	0.000352609	2836
PMM2	NM_000303.2:c.674del(p.Arg225Lysfs*7)	0.000352609	2836
PMM2	NM_000303.2:c.1A>G(p.Met1?)	0.000352609	2836
PMM2	NM_000303.2:c.527G>T(p.Gly176Val)	0.000352609	2836

ACADM	NM_000016.5:c.535_538dup(p.Gly180Glufs*4)	0.000352609	2836
ACADM	NM_000016.5:c.745G>T(p.Gly249*)	0.000352609	2836
ACADM	NM_000016.5:c.617G>A(p.Arg206His)	0.000352609	2836
ACADM	NM_000016.5:c.1189T>A(p.Tyr397Asn)	0.000352609	2836
ACADM	NM_000016.5:c.1085G>A(p.Gly362Glu)	0.000352609	2836
ATP7B	NM_000053.3:c.1517_1518del(p.Ile506Argfs*27)	0.000352609	2836
ATP7B	NM_000053.3:c.1531C>T(p.Gln511*)	0.000352609	2836
ATP7B	NM_000053.3:c.2294A>G(p.Asp765Gly)	0.000352609	2836
ATP7B	NM_000053.3:c.2804C>T(p.Thr935Met)	0.000352609	2836
ATP7B	NM_000053.3:c.4114C>T(p.Gln1372*)	0.000352609	2836
ATP7B	NM_000053.3:c.3532A>G(p.Thr1178Ala)	0.000352609	2836
ATP7B	NM_000053.3:c.2304dup(p.Met769Hisfs*26)	0.000352609	2836
ATP7B	NM_000053.3:c.1708-1G>C	0.000352609	2836
ATP7B	NM_000053.3:c.2183A>G(p.Asn728Ser)	0.000352609	2836
ATP7B	NM_000053.3:c.1708-5T>G	0.000352609	2836
ATP7B	NM_000053.3:c.2549C>T(p.Thr850Ile)	0.000352609	2836
ATP7B	NM_000053.3:c.3182G>A(p.Gly1061Glu)	0.000352609	2836
ATP7B	NM_000053.3:c.3646G>A(p.Val1216Met)	0.000352609	2836
ATP7B	NM_000053.3:c.3809A>G(p.Asn1270Ser)	0.000352609	2836
ASS1	NM_000050.4:c.421-2A>G	0.000352609	2836
ASS1	NM_000050.4:c.431C>G(p.Pro144Arg)	0.000352609	2836
ASS1	NM_000050.4:c.787G>A(p.Val263Met)	0.000352609	2836
GLDC	NM_000170.2:c.1718G>A(p.Trp573*)	0.000352609	2836
PC	NM_000920.3:c.3289-1G>A	0.000352609	2836
PC	NM_000920.3:c.903+1G>A	0.000352609	2836
NPC1	NM_000271.4:c.3182T>C(p.Ile1061Thr)	0.000352609	2836
NPC1	NM_000271.4:c.1800del(p.Ile601Phefs*13)	0.000352609	2836
NPC1	NM_000271.4:c.3734_3735del(p.Pro1245Argfs*12)	0.000352609	2836
NPC1	NM_000271.4:c.2373+2T>C	0.000352609	2836
NPC1	NM_000271.4:c.2903A>G(p.Asn968Ser)	0.000352609	2836
NPC1	NM_000271.4:c.3477+2T>C	0.000352609	2836
SLC35A1	NM_006416.4:c.16+1G>A	0.000352609	2836
PTS	NM_000317.2:c.317C>T(p.Thr106Met)	0.000352609	2836
AMT	NM_000481.3:c.15_18del(p.Ser6Trpfs*89)	0.000352609	2836
DLD	NM_000108.4:c.882del(p.Ala295Leufs*30)	0.000352609	2836
SLC26A2	NM_000112.3:c.611T>G(p.Leu204*)	0.000352609	2836
COL4A4	NM_000092.4:c.282_283del(p.Asp96Profs*13)	0.000352609	2836
COL4A4	NM_000092.4:c.42del(p.Arg14Serfs*2)	0.000352609	2836
COL4A4	NM_000092.4:c.1099+2T>C	0.000352609	2836
SLC26A4	NM_000441.1:c.2162C>T(p.Thr721Met)	0.000352609	2836
SLC26A4	NM_000441.1:c.1975G>C(p.Val659Leu)	0.000352609	2836
SLC26A4	NM_000441.1:c.1594A>C(p.Ser532Arg)	0.000352609	2836
SLC26A4	NM_000441.1:c.918+2T>C	0.000352609	2836
SLC26A4	NM_000441.1:c.2027T>A(p.Leu676Gln)	0.000352609	2836
SLC26A4	NM_000441.1:c.1181_1183del(p.Phe394del)	0.000352609	2836
SLC26A4	NM_000441.1:c.281C>T(p.Thr94Ile)	0.000352609	2836
SLC26A4	NM_000441.1:c.2009T>C(p.Val670Ala)	0.000352609	2836
SLC26A4	NM_000441.1:c.919-2A>_G	0.000352609	2836
SLC26A4	NM_000441.1:c.563T>C(p.Ile188Thr)	0.000352609	2836
SLC26A4	NM_000441.1:c.1574C>T(p.Pro525Leu)	0.000352609	2836
SLC26A4	NM_000441.1:c.349del(p.Leu117Serfs*9)	0.000352609	2836
SLC26A4	NM_000441.1:c.916dup(p.Val306Glyfs*24)	0.000352609	2836
NPHS1	NM_004646.3:c.3482-1G>A	0.000352609	2836
ETHE1	NM_014297.4:c.488G>A(p.Arg163Gln)	0.000352609	2836
ETHE1	NM_014297.4:c.79C>T(p.Gln27*)	0.000352609	2836

IVD	NM_002225.3:c.466-3_466-2delinsGG	0.000352609	2836
ABCA12	NM_173076.2:c.6167dup(p.Lys2057Glnfs*8)	0.000352609	2836
PEX6	NM_000287.3:c.2666+1G>C	0.000352609	2836
PEX6	NM_000287.3:c.2037delT(p.Phe679Leufs*35)	0.000352609	2836
POMGNT1	NM_017739.3:c.511C>T(p.Arg171*)	0.000352609	2836
POMGNT1	NM_017739.3:c.667G>A(p.Glu223Lys)	0.000352609	2836
POMGNT1	NM_017739.3:c.296T>C(p.Leu99Pro)	0.000352609	2836
POMGNT1	NM_017739.3:c.931C>T(p.Arg311*)	0.000352609	2836
POMGNT1	NM_017739.3:c.1285-2A>G	0.000352609	2836
MOGS	NM_006302.2:c.1483C>T(p.Arg495*)	0.000352609	2836
MOGS	NM_006302.2:C.195G>A(p.Trp65*)	0.000352609	2836
MOGS	NM_006302.2(MOGS):c.1212_1239dup(p.Asp414Leufs*17)	0.000352609	2836
G6PC	NM_000151.3:c.279C>A(p.Tyr93*)	0.000352609	2836
G6PC	NM_000151.3:c.518T>C(p.Leu173Pro)	0.000352609	2836
G6PC	NM_000151.3:c.356A>T(p.His119Leu)	0.000352609	2836
G6PC	NM_000151.3:c.310C>T(p.Gln104*)	0.000352609	2836
G6PC	NM_000151.3:c.248G>A(p.Arg83His)	0.000352609	2836
ABCB11	NM_003742.2:c.1493T>C(p.Ile498Thr)	0.000352609	2836
ABCB11	NM_003742.2:c.3593A>G(p.His1198Arg)	0.000352609	2836
DBT	NM_001918.3:c.75_76del(p.Cys26Trpfs*2)	0.000352609	2836
CYP27A1	NM_000784.3:c.1435C>T(p.Arg479Cys)	0.000352609	2836
CYP27A1	NM_000784.3:c.446+1G>T	0.000352609	2836
CYP27A1	NM_000784.3:c.409C>T(p.Arg137Trp)	0.000352609	2836
CYP27A1	NM_000784.3:c.1016C>T(p.Thr339Met)	0.000352609	2836
CYP27A1	NM_000784.3:c.380G>A(p.Arg127Gln)	0.000352609	2836
CYP27A1	NM_000784.3:c.1072C>T(p.Gln358Ter)	0.000352609	2836
CYP27A1	NM_000784.3:c.562C>T(p.Arg188*)	0.000352609	2836
SLC22A5	NM_003060.3:c.1433C>T(p.Pro478Leu)	0.000352609	2836
SLC22A5	NM_003060.3:c.797C>T(p.Pro266Leu)	0.000352609	2836
SLC22A5	NM_003060.3:c.209_240dup(p.Cys81Glyfs*60)	0.000352609	2836
MMACHC	NM_015506.2:c.217C>T(p.Arg73*)	0.000352609	2836
MMACHC	NM_015506.2:c.445_446del(p.Cys149Hisfs*32)	0.000352609	2836
MMACHC	NM_015506.2:c.688C>T(p.Arg230*)	0.000352609	2836
MMACHC	NM_015506.2:c.626dup(p.Thr210Aspfs*35)	0.000352609	2836
MMACHC	NM_015506.2:c.394C>T(p.Arg132*)	0.000352609	2836
MMACHC	NM_015506.2:c.1A>G(p.Met1?)	0.000352609	2836
MMACHC	NM_015506.2:c.315C>G(p.Tyr105*)	0.000352609	2836
PCDH15	NM_033056.3:c.4970_4971dup(p.Ser1658Leufs*187)	0.000352609	2836
PCDH15	NM_033056.3:c.1997+1G>C	0.000352609	2836
PCDH15	NM_033056.3:c.1006C>T(p.Arg336*)	0.000352609	2836
PCDH15	NM_033056.3:c.4533_4554dup(p.Glu1519Lysfs*7)	0.000352609	2836
POMT1	NM_007171.3:c.542del(p.Pro181Leufs*4)	0.000352609	2836
POMT1	NM_007171.3:c.897del(p.Ser300Valfs*7)	0.000352609	2836
POMT1	NM_007171.3:c.1731del(p.Asn578Ilefs*20)	0.000352609	2836
POMT1	NM_007171.3:c.605+1G>A	0.000352609	2836
POMT1	NM_007171.3:c.1457G>C(p.Trp486Ser)	0.000352609	2836
POMT1	NM_007171.3:c.2005G>A(p.Ala669Thr)	0.000352609	2836
COL4A3	NM_000091.4:c.2417dup(p.Gly807Argfs*28)	0.000352609	2836
POMT2	NM_013382.5(POMT2):c.495_522delins(p.Asp165Glufs*12)	0.000352609	2836
POMT2	NM_013382.5:c.1237C>T(p.Arg413*)	0.000352609	2836
POMT2	NM_013382.5:c.2033-1G>C	0.000352609	2836
POMT2	NM_013382.5:c.1261C>T(p.Arg421Trp)	0.000352609	2836
NCF2	NM_000433.3:c.574C>T(p.Gln192*)	0.000352609	2836
NCF2	NM_000433.3:c.304C>T(p.Arg102*)	0.000352609	2836
GNS	NM_002076.3:c.1229_1233del(p.Ser410Cysfs*11)	0.000352609	2836

PROP1	NM_006261.4:c.46C>T(p.Arg16*)	0.000352609	2836
ATM	NM_000051.3:c.5679_5691del(p.Glu1894Aspfs*19)	0.000352609	2836
AHI1	NM_017651.4:c.910dup(p.Thr304Asnfs*6)	0.000352609	2836
AHI1	NM_017651.4:c.1992del(p.Asp665Metfs*21)	0.000352609	2836
AHI1	NM_017651.4:c.379A>T(p.Lys127*)	0.000352609	2836
AHI1	NM_017651.4:c.2105C>T(p.Thr702Met)	0.000352609	2836
POLG	NM_002693.2:c.3218C>T(p.Pro1073Leu)	0.000352609	2836
POLG	NM_002693.2:c.3139C>T(p.Arg1047Trp)	0.000352609	2836
SMPD1	NM_000543.4:c.1805G>A(p.Arg602His)	0.000352609	2836
SMPD1	NM_000543.4:c.557C>T(p.Pro186Leu)	0.000352609	2836
SMPD1	NM_000543.4:c.203_205delinsAGGGGAGA(p.Gly68Glufs*11)	0.000352609	2836
SMPD1	NM_000543.4:c.1118dup(p.Tyr374Ilefs*17)	0.000352609	2836
HGSNAT	NM_152419.2:c.1345dup(p.Asp449Glyfs*21)	0.000352609	2836
HGSNAT	NM_152419.2:c.1030C>T(p.Arg344Cys)	0.000352609	2836
HGSNAT	NM_152419.2:c.493+1G>A	0.000352609	2836
CHAT	NM_020549.4:c.1618C>T(p.Gln540*)	0.000352609	2836
CHAT	NM_020549.4:c.243G>A(p.Trp81*)	0.000352609	2836
CHAT	NM_020549.4:c.1095del(p.Thr366Argfs*4)	0.000352609	2836
CHAT	NM_020549.4:c.1061C>T(p.Thr354Met)	0.000352609	2836
FKTN	NM_001079802.1:c.1117G>T(p.Glu373*)	0.000352609	2836
FKTN	NM_001079802.1:c.1106del(p.Phe369Serfs*37)	0.000352609	2836
FKTN	NM_001079802.1:c.139C>T(p.Arg47*)	0.000352609	2836
HEXB	NM_000521.3:c.1069G>T(p.Glu357*)	0.000352609	2836
HEXB	NM_000521.3:c.1263_1268del(p.Glu422_Val423del)	0.000352609	2836
HEXB	NM_000521.3:c.257_268delinsGC(p.Thr86Serfs*65)	0.000352609	2836
HEXB	NM_000521.3:c.118del(p.Ala40Argfs*24)	0.000352609	2836
HEXB	NM_000521.3:c.1250C>T(p.Pro417Leu)	0.000352609	2836
ALDOB	NM_000035.3:c.43del(p.Glu15Serfs*23)	0.000352609	2836
ALDOB	NM_000035.3:c.360_363del(p.Asn120Lysfs*32)	0.000352609	2836
ALDOB	NM_000035.3:c.10C>T(p.Arg4*)	0.000352609	2836
ALDOB	NM_000035.3:c.673del(p.Glu225Argfs*5)	0.000352609	2836
ALDOB	NM_000035.3:c.888G>A(p.Trp296*)	0.000352609	2836
CYP1B1	NM_000104.3:c.1090G>A(p.Val364Met)	0.000352609	2836
CLN6	NM_017882.2:c.316dup(p.Arg106Profs*26)	0.000352609	2836
SGCG	NM_000231.2:c.768del(p.Ser257Alafs*23)	0.000352609	2836
SGCA	NM_000023.2:c.229C>T(p.Arg77Cys)	0.000352609	2836
ACAT1	NM_000019.3:c.1117A>T(p.Lys373*)	0.000352609	2836
FANCC	NM_000136.2:c.1417C>T(p.Gln473*)	0.000352609	2836
FANCC	NM_000136.2:c.843+1G>A	0.000352609	2836
FANCC	NM_000136.2:c.125dup(p.Glu43Glyfs*8)	0.000352609	2836
FANCC	NM_000136.2:c.339G>A(p.Trp113*)	0.000352609	2836
NBN	NM_002485.4:c.1192C>T(p.Gln398*)	0.000352609	2836
NBN	NM_002485.4:c.1882_1885del(p.Glu628Thrfs*28)	0.000352609	2836
LYST	NM_000081.3:c.3232del(p.Val1078Phefs*65)	0.000352609	2836
LYST	NM_000081.3:c.9874G>T(p.Glu3292*)	0.000352609	2836
LYST	NM_000081.3:c.10349_10353del(p.Glu3450Glyfs*67)	0.000352609	2836
DPM1	NM_003859.1:c.571C>T(p.Arg191*)	0.000352609	2836
DPM1	NM_003859.1:c.46G>T(p.Glu16*)	0.000352609	2836
DPM1	NM_003859.1:c.564-2A>G	0.000352609	2836
ASL	NM_000048.3:c.291+1G>T	0.000352609	2836
GALT	NM_000155.3:c.610C>T(p.Arg204*)	0.000352609	2836
PEX1	NM_000466.2:c.2966T>C(p.Ile989Thr)	0.000352609	2836
PEX1	NM_000466.2:c.643_647del(p.Thr215Alafs*11)	0.000352609	2836
PEX1	NM_000466.2:c.2614C>T(p.Arg872*)	0.000352609	2836
PEX1	NM_000466.2:c.2686C>T(p.Arg896*)	0.000352609	2836

PEX1	NM_000466.2:c.1926_1927delinsC(p.Lys642Asnfs*3)	0.000352609	2836
PEX1	NM_000466.2:c.357+1G>T	0.000352609	2836
PEX1	NM_000466.2:c.1803+1G>A	0.000352609	2836
PEX1	NM_000466.2:c.960del(p.Glu320Aspfs*5)	0.000352609	2836
PEX1	NM_000466.2:c.1671_1672del(p.Gly558Serfs*33)	0.000352609	2836
PEX1	NM_000466.2:c.2692_2693del(p.Ser898*)	0.000352609	2836
PEX1	NM_000466.2:c.1A>T(p.Met1?)	0.000352609	2836
ETFB	NM_001985.2:c.2T>C	0.000352609	2836
QDPR	NM_000320.2:c.484C>T(p.Gln162*)	0.000352609	2836
NAGLU	NM_000263.3:c.1041G>A(p.Trp347*)	0.000352609	2836
NAGLU	NM_000263.3:c.679-5_679-1del	0.000352609	2836
MMUT	NM_000255.3:c.1038_1040del(p.Leu347del)	0.000352609	2836
MMUT	NM_000255.3:c.1782_1786del(p.Ser594Argfs*11)	0.000352609	2836
MMUT	NM_000255.3:c.729_730insTT(p.Asp244Leufs*39)	0.000352609	2836
MMUT	NM_000255.3:c.349G>T(p.Glu117*)	0.000352609	2836
MMUT	NM_000255.3:c.914T>C(p.Leu305Ser)	0.000352609	2836
MMUT	NM_000255.3:c.1105C>T(p.Arg369Cys)	0.000352609	2836
MMUT	NM_000255.3:c.755dup(p.His252Glnfs*6)	0.000352609	2836
MMUT	NM_000255.3:c.1615G>T(p.Glu539*)	0.000352609	2836
MMUT	NM_000255.3:c.2179C>T(p.Arg727*)	0.000352609	2836
MMUT	NM_000255.3:c.1677-1G>A	0.000352609	2836
UNC13D	NM_199242.2:c.3193C>T(p.Arg1065*)	0.000352609	2836
UNC13D	NM_199242.2:c.640C>T(p.Arg214*)	0.000352609	2836
UNC13D	NM_199242.2:c.706del(p.Asp236Thrfs*13)	0.000352609	2836
UNC13D	NM_199242.2:c.1596+1G>C	0.000352609	2836
UNC13D	NM_199242.2:C.1597-2A>T	0.000352609	2836
HBA1/HBA2	heterozygous CFIL/THAI deletion	0.000352609	2836
HBA1/HBA2	HBA2 heterozygous	0.000352609	2836
GALC	NM_000153.3:c.956A>G(p.Tyr319Cys)	0.000352609	2836
GALC	NM000153.3:c.1901T>C(p.Leu634Ser)	0.000352609	2836
GALC	NM_000153.3:c.1321C>T(p.Gln441*)	0.000352609	2836
GALC	NM_000153.3:c.467_468dup(p.Leu157Glyfs*15)	0.000352609	2836
GALC	NM_000153.3:c.1054C>T(p.Gln352*)	0.000352609	2836
GALC	NM_000153.3:c.908+1G>C	0.000352609	2836
GALC	NM_000153.3:c.461C>A(p.Pro154His)	0.000352609	2836
BTD	NM_000060.4:c.1493dup(p.Leu498Phefs*13)	0.000352609	2836
BTD	NM_000060.4:c.470G>A(p.Arg157His)	0.000352609	2836
BTD	NM_000060.4:c.1A>G(p.Met1?)	0.000352609	2836
BTD	NM_000060.4:c.494dup(p.Asp166Argfs*16)	0.000352609	2836
IDUA	NM_000203.4:c.1323C>A(p.Tyr441*)	0.000352609	2836
IDUA	NM_000203.4:c.530dup(p.Glu178Argfs*5)	0.000352609	2836
IDUA	NM_000203.4:c.1422_1423dup(p.Tyr475Serfs*51)	0.000352609	2836
IDUA	NM_000203.4:c.1210G>T(p.Glu404*)	0.000352609	2836
IDUA	NM_000203.4:c.1402+2T>G	0.000352609	2836
AGXT	NM_000030.2:c.32C>G(p.Pro11Arg)	0.000352609	2836
AGXT	NM_000030.2:c.1079G>A(p.Arg360Gln)	0.000352609	2836
AGXT	NM_000030.2:c.569_570dup(p.Thr191Glyfs*22)	0.000352609	2836
AGXT	NM_000030.2:c.823_824dup(p.Ser275Argfs*38)	0.000352609	2836
AGXT	NM_000030.2:c.1161C>A(p.Cys387*)	0.000352609	2836
AGXT	NM_000030.2:c.904del(p.Leu302Cysfs*10)	0.000352609	2836
GLB1	NM_000404.3:c.245C>T(p.Thr82Met)	0.000352609	2836
GLB1	NM_000404.3:c.1838del(p.Pro613Glnfs*24)	0.000352609	2836
GLB1	NM_000404.2:c.75+2dup	0.000352609	2836
GLB1	NM_000404.2:c.1143+1G>A	0.000352609	2836
BBS1	NM_024649.4:c.479G>A(p.Arg160Gln)	0.000352609	2836

ADA	NM_000022.3:c.606+1G>T	0.000352609	2836
SGSH	NM_000199.4:c.1130G>A(p.Arg377His)	0.000352609	2836
SGSH	NM_000199.4:c.398del(p.Pro133Argfs*131)	0.000352609	2836
SGSH	NM_000199.3:c.858C>G(p.Tyr286*)	0.000352609	2836
HLCS	NM_000411.7:c.782del(p.Gly261Valfs*20)	0.000352609	2836
HLCS	NM_000411.6:c.1522C>T(p.Arg508Trp)	0.000352609	2836
HLCS	NM_000411.7:c.1544G>A(p.Ser515Asn)	0.000352609	2836
HLCS	NM_000411.6:c.782del(p.Gly261Valfs*20)	0.000352609	2836
HLCS	NM_000411.7:c.1711G>A(p.Asp571Asn)	0.000352609	2836
GUSB	NM_000181.3:c.279del(p.His94Ilefs*12)	0.000352609	2836
GUSB	NM_000181.3:c.715C>T(p.Gln239*)	0.000352609	2836
GUSB	NM_000181.3:c.91C>T(p.Gln31*)	0.000352609	2836
MPL	NM_005373.2:c.127C>T(p.Arg43*)	0.000352609	2836
MPL	NM_005373.2:c.235_236del(p.Leu79Glufs*84)	0.000352609	2836
MPL	NM_005373.2:c.1413del(p.Gly472Valfs*24)	0.000352609	2836
MPL	NM_005373.2:c.1468+1G>A	0.000352609	2836
ETFA	NM_000126.3:c.665-1G>A	0.000352609	2836
ETFA	NM_000126.3:c.619del(p.Ser207Valfs*6)	0.000352609	2836
HAX1	NM_006118.3:c.430dupG(p.Val144Glyfs*5)	0.000352609	2836
PLOD1	NM_000302.3:c.1471-2A>G	0.000352609	2836
PLOD1	NM_000302.3:c.976-1G>A	0.000352609	2836
PLOD1	NM_000302.3:c.607del(p.Arg203Alafs*24)	0.000352609	2836
PLOD1	NM_000302.3:c.2107C>T(p.Arg703*)	0.000352609	2836
GNE	NM_001128227.2:c.830G>A(p.Arg277Gln)	0.000352609	2836
GNE	NM001128227.2:c.18T>A(p.Tyr6*)	0.000352609	2836
MPI	NM_002435.2:c.1A>G(p.Met1?)	0.000352609	2836
GCDH	NM_000159.3:c.892G>A(p.Ala298Thr)	0.000352609	2836
GCDH	NM_000159.3:c.533G>A(p.Gly178Glu)	0.000352609	2836
GCDH	NM_000159.3:c.1213A>G(p.Met405Val)	0.000352609	2836
GCDH	NM_000159.3:c.356C>T(p.Ser119Leu)	0.000352609	2836
GCDH	NM_000159.3:c.383G>A(p.Arg128Gln)	0.000352609	2836
GCDH	NM_000159.3:c.1244-2A>	0.000352609	2836
GCDH	NM_000159.3:c.572T>C(p.Met191Thr)	0.000352609	2836
GCDH	NM_000159.3:c.413G>A(p.Arg138Lys)	0.000352609	2836
GCDH	NM_000159.3:c.532G>A(p.Gly178Arg)	0.000352609	2836
SERPINA1	NM_000295.4:c.1096G>A(p.Glu366Lys)	0.000352609	2836
SLC37A4	NM_001164277.1:c.1243C>T(p.Arg415*)	0.000352609	2836
SLC37A4	NM_001164277.1:c.354del(p.Trp118Cysfs*28)	0.000352609	2836
SLC37A4	NM_001164277.1:c1042_1043del(p.Leu348Valfs*53)	0.000352609	2836
SLC37A4	NM_001164277.1:c.1042_1043del(p.Leu348Valfs*53)	0.000352609	2836
SLC37A4	NM_001164277.1:c.572C>T(p.Pro191Leu)	0.000352609	2836
DPYD	NM_000110.3:c.197del(p.Thr66Ilefs*12)	0.000352609	2836
DPYD	NM_000110.3:c.1054del(p.Leu352Tyrfs*19)	0.000352609	2836
HADHB	NM_000183.2:c.940_942delinsTAA(p.Gly314*)	0.000352609	2836
GJB2	NM_004004.5:c.35dup(p.Val13Cysfs*35)	0.000352609	2836
GJB2	NM_004004.5:c.230G>A(p.Trp77*)	0.000352609	2836
GJB2	NM_004004.5(GJB2):c.235del(p.Leu79Cysfs*3)	0.000352609	2836
GJB2	NM_004004.5:c.238C>T(p.Gln80*)	0.000352609	2836
GJB2	NM_004004.5:c.279G>A(p.Met93Ile)	0.000352609	2836
FAH	NM_000137.2:c.520C>T(p.Arg174*)	0.000352609	2836
GBE1	NM_000158.3:c.1544G>A(p.Arg515His)	0.000352609	2836
GBE1	NM_000158.3:c.288del(p.Gly97Glufs*46)	0.000352609	2836
TH	NM_199292.2:c.1136C>A(p.Ser379*)	0.000352609	2836
TH	NM_199292.2:c.580+2T>C	0.000352609	2836
TH	NM_199292.2:c.739G>A(p.Gly247Ser)	0.000352609	2836

TH	NM_199292.2:c.1128_1138del(p.Gln377Glyfs*12)	0.000352609	2836
ABCC8	NM_000352.4:c.331G>A(p.Gly111Arg)	0.000352609	2836
ABCC8	NM_000352.4:c.3541_3542del(p.Phe1181Profs*12)	0.000352609	2836
ABCC8	NM_000352.4:c.3989-9G>A	0.000352609	2836
TTPA	NM_000370.3:c.358G>A(p.Ala120Thr)	0.000352609	2836
GAA	NM_000152.4:c.546+5G>T	0.000352609	2836
GAA	NM_000152.3:c.1978C>T(p.Arg660Cys)	0.000352609	2836
GAA	NM_000152.4:c.2238G>C(p.Trp746Cys)	0.000352609	2836
GAA	NM_000152.3:c.1822C>T(p.Arg608*)	0.000352609	2836
GAA	NM_000152.3:c.796C>T(p.Pro266Ser)	0.000352609	2836
GAA	NM_000152.3:c.784G>A(p.Glu262Lys)	0.000352609	2836
GAA	NM_000152.3:c.2238G>C(p.Trp746Cys)	0.000352609	2836
GAA	NM_000152.4:c.2185del(p.Leu729Trpfs*35)	0.000352609	2836
GAA	NM_000152.3:c.1692del(p.Leu565Serfs*13)	0.000352609	2836
GAA	NM_000152.4:c.169C>T(p.Gln57*)	0.000352609	2836
GAA	NM_000152.4:c.1316T>A(p.Met439Lys)	0.000352609	2836
GAA	NM_000152.4:c.546G>A(p.Thr182=)	0.000352609	2836
GAA	NM_000152.4:c.1822C>T(p.Arg608*)	0.000352609	2836
GAA	NM_000152.3:c.1634C>T(p.Pro545Leu)	0.000352609	2836
GAA	NM_000152.3:c.2185del(p.Leu729Trpfs*35)	0.000352609	2836
GAA	NM_000152.4:c.1320_1322del(p.Met440del)	0.000352609	2836
GAA	NM_000152.3:c.543del(p.Phe181Leufs*40)	0.000352609	2836
GAA	NM_000152.4:c.1551+2T>G	0.000352609	2836
GAA	NM_000152.4:c.1979G>A(p.Arg660His)	0.000352609	2836
MFSD8	NM_152778.2:c.439+2T>A	0.000352609	2836
MFSD8	NM_152778.2:c.1351-1G>A	0.000352609	2836
SBDS	NM016038.2:c.258+2T>C	0.000352609	2836
SBDS	NM_016038.2:c.258+2T>C	0.000352609	2836
SBDS	NM_016038.3:c.454C>T(p.Gln152*)	0.000352609	2836
TPP1	NM_000391.3:c.157_158del(p.Leu53Glufs*34)	0.000352609	2836
TPP1	NM_000391.3:c.1A>G(p.Met1?)	0.000352609	2836
BCKDHA	NM_000709.3:c.632C>T(p.Thr211Met)	0.000352609	2836
BCKDHA	NM_000709.3:c.117dup(p.Arg40Glnfs*11)	0.000352609	2836
CYBA	NM_000101.3:c.27G>A(p.Trp9*)	0.000352609	2836
MCOLN1	NM_020533.2:c.1426_1427del(p.Thr476Valfs*131)	0.000352609	2836
HBA2	NM_000517.4:c.377T>C(p.Leu126Pro)	0.000352609	2836
HBA2	NM_000517.4:c.427T>C(p.*143Glnext*31)	0.000352609	2836
CTNS	NM_004937.2:c.969C>G(p.Asn323Lys)	0.000352609	2836
CTNS	NM_004937.2:c.589G>A(p.Gly197Arg)	0.000352609	2836
CTNS	NM_004937.2:c.1015G>A(p.Gly339Arg)	0.000352609	2836
ARSA	NM_000487.5:c.465G>A(p.Gln155=)	0.000352609	2836
ARSA	NM_000487.5:c.302G>T(p.Gly101Val)	0.000352609	2836
ARSA	NM_000487.5:c.185_186dup(p.Asp63Glnfs*18)	0.000352609	2836
ARSA	NM_000487.5:c.257G>A(p.Arg86Gln)	0.000352609	2836
ARSA	NM_000487.5:c.1108-3C>G	0.000352609	2836
ARSB	NM_000046.4:c.1507C>T(p.Gln503*)	0.000352609	2836
ARSB	NM_000046.4:c.571C>T(p.Arg191*)	0.000352609	2836
ARSB	NM_000046.4:c.1197C>G(p.Phe399Leu)	0.000352609	2836
ARSB	NM_000046.3:c.1197C>G(p.Phe399Leu)	0.000352609	2836
HEXA	NM_000520.4:c.745C>T(p.Arg249Trp)	0.000352609	2836
HEXA	NM_000520.5:c.546dup(p.Leu183Thrfs*3)	0.000352609	2836
HEXA	NM_000520.4:c.546dup(p.Leu183Thrfs*3)	0.000352609	2836
HEXA	NM_000520.4:c.1142_1146del(p.Val381Aspfs*48)	0.000352609	2836
BBS2	NM_031885.3:c.1525_1526del(p.Arg509Glyfs*42)	0.000352609	2836
BBS2	NM_031885.3:c.1725dup(p.Ala576Cysfs*3)	0.000352609	2836

BBS2	NM_031885.3:c.1059dup(p.Asn354*)	0.000352609	2836
BBS2	NM_031885.3:c.2107C>T(p.Arg703*)	0.000352609	2836
BBS2	NM_031885.3:c.700C>T(p.Arg234*)	0.000352609	2836
BBS2	NM_031885.3:c.563delT(p.Ile188Thrfs*13)	0.000352609	2836
RAPSN	NM_005055.4:c.288delG(p.Cys97Alafs*31)	0.000352609	2836
RAPSN	NM_005055.4:c.39del(p.Leu14Serfs*50)	0.000352609	2836
MLC1	NM_015166.3:c.353C>T(p.Thr118Met)	0.000352609	2836
BCKDHB	NM_000056.3:c.548G>C(p.Arg183Pro)	0.000352609	2836
BCKDHB	NM_183050.3:c.550del(p.Ser184Profs*46)	0.000352609	2836
BCKDHB	NM_183050.2:c.332del(p.Arg111Glnfs*119)	0.000352609	2836
BCKDHB	NM_183050.2:c.409dup(p.Ala137Glyfs*22)	0.000352609	2836
CBS	NM_000071.2:c.19dup(p.Gln7Profs*30)	0.000352609	2836
CHRNE	NM_000080.3:c.1203del(p.Gln402Argfs*26)	0.000352609	2836
MYO7A	NM_000260.3:c.5892del(p.Phe1965Leufs*5)	0.000352609	2836
MYO7A	NM_000260.3:c.5581C>T(p.Arg1861*)	0.000352609	2836
MYO7A	NM_000260.3:c.721C>T(p.Arg241Cys)	0.000352609	2836
MYO7A	NM_000260.3:c.3412C>T(p.Gln1138*)	0.000352609	2836
MYO7A	NM_000260.3:c.5648G>A(p.Arg1883Gln)	0.000352609	2836
MYO7A	NM_000260.3(MYO7A):c.2476G>A(p.Ala826Thr)	0.000352609	2836
MYO7A	NM_000260.3:c.1798-1G>C	0.000352609	2836
MYO7A	NM_000260.3:c.3050dup(p.Tyr1017*)	0.000352609	2836
ALPL	NM_000478.4(ALPL):c.997+1G>T	0.000352609	2836
ALPL	NM_000478.5:c.1190-2A>T	0.000352609	2836
ALPL	NM_000478.4:c.979T>C(p.Phe327Leu)	0.000352609	2836
ALPL	NM_000478.5:c.1471G>A(p.Gly491Arg)	0.000352609	2836
