# Supplementary material for: Expanded carrier screening in Chinese patients seeking the help of assisted reproductive technology
Source: Mol Genet Genomic Med. 2020 Jun 23;8(9):e1340. doi: 10.1002/mgg3.1340 (PMC7507411; doi:10.1002/mgg3.1340)
Supplement: Supplementary file 5 — Table S5 [file MGG3-8-e1340-s005.pdf]

Table S5. The gene carrier rates (GCRs) estimated from the data of 2,836 Han Chinese individuals with no family history across the 187 AR genes covered by the ECS test.

gene_symbol	GCR	1 in _
SLC25A13	0.038681257	25.85231397
GJB2	0.036649183	27.28573763
GALC	0.027382412	36.51979231
USH2A	0.02649276	37.74616148
ATP7B	0.023037492	43.40750355
SLC26A4	0.022028721	45.39528291
HBA1/HBA2	0.021388273	46.75459333
PAH	0.019215516	52.0412764
SLC22A5	0.015440383	64.76523403
CYP1B1	0.0140861	70.99197035
SMN1	0.011988717	83.41176471
TYR	0.011923411	83.86862073
PMM2	0.011584013	86.32586927
PKHD1	0.01122722	89.06924522
GAA	0.010874501	91.9582409
MLC1	0.010574674	94.56556654
MMACHC	0.009834598	101.6818328
ETFDH	0.009487686	105.3997818
GNE	0.009140027	109.4088647
CYP27A1	0.008431606	118.6013695
CFTR	0.00807987	123.7643708
CAPN3	0.007393736	135.2496141
MMUT	0.007380597	135.490394
G6PC	0.006686658	149.551539
COL4A3	0.006344854	157.6080422
ALPL	0.006329712	157.985079
UNC13D	0.005633673	177.5041048
HBB	0.00528007	189.3914283
COL7A1	0.0052766	189.5159867
CEP290	0.005276228	189.5293263
CDH23	0.004932056	202.7552075
PTS	0.004928454	202.9034029
SMPD1	0.004576963	218.4854727
OCA2	0.004574979	218.5802464
PEX1	0.004574359	218.6098453
DPYD	0.004225719	236.6461047
GCDH	0.004223487	236.7711732
PRF1	0.003872491	258.2317118
AHI1	0.003872491	258.2317118
ACADS	0.003872367	258.2399768
ACADVL	0.003871995	258.2647808
ACADM	0.003521744	283.9502205
ALDOB	0.003521744	283.9502205
MYO7A	0.003520627	284.0402878
GALT	0.003172489	315.2099075
BBS2	0.003169384	315.5187624
GNPTAB	0.003169384	315.5187711
BTD	0.00281814	354.8439291
TH	0.00281814	354.8439291
NPHS1	0.002818016	354.8595851

AGXT	0.00281752	354.9221016
ALDH3A2	0.002466898	405.3674354
SLC45A2	0.002466152	405.4899707
SGSH	0.002465904	405.5308035
ARSA	0.00246578	405.5512158
DHCR7	0.002465656	405.5716301
MOGS	0.002114164	473.0001372
SBDS	0.002114164	473.0001372
PCCB	0.002113916	473.0557188
NPHS2	0.002113916	473.0557188
PCDH15	0.002113916	473.0557188
NPC1	0.002113792	473.0835048
POMT1	0.002113792	473.0835048
TGM1	0.001762549	567.3600451
CPT2	0.001762176	567.4800959
HBA2	0.001762176	567.4800959
RAPSN	0.001762176	567.4800959
GRHPR	0.001762052	567.5201242
IVD	0.001762052	567.5201242
SERPINA1	0.001762052	567.5201242
AGL	0.001761804	567.6001411
PLA2G6	0.001761804	567.6001411
PYGM	0.001761804	567.6001411
POMGNT1	0.001761804	567.6001411
HEXB	0.001761804	567.6001411
IDUA	0.001761804	567.6001411
HLCS	0.001761804	567.6001411
SLC37A4	0.001761804	567.6001411
SLC35A1	0.001410064	709.1875496
MMAA	0.001409816	709.3125937
PCCA	0.001409691	709.3751102
DOK7	0.001409691	709.3751102
LIPA	0.001409691	709.3751102
POMT2	0.001409691	709.3751102
CHAT	0.001409691	709.3751102
FANCC	0.001409691	709.3751102
GLB1	0.001409691	709.3751102
PLOD1	0.001409691	709.3751102
MPL	0.001409691	709.3751102
ARSB	0.001409691	709.3751102
HEXA	0.001409691	709.3751102
BCKDHB	0.001409691	709.3751102
ACAT1	0.001057579	945.5556078
ADA	0.001057579	945.5556078
HAX1	0.001057579	945.5556078
HADHB	0.001057579	945.5556078
CYBA	0.001057579	945.5556078
CBS	0.001057579	945.5556078
CLN5	0.001057455	945.666745
SLC12A6	0.001057455	945.666745
SACS	0.001057455	945.666745
ABCC8	0.001057455	945.666745
ASS1	0.001057455	945.666745

COL4A4	0.001057455	945.666745
BBS10	0.001057455	945.666745
HGSNAT	0.001057455	945.666745
FKTN	0.001057455	945.666745
LYST	0.001057455	945.666745
DPM1	0.001057455	945.666745
GUSB	0.001057455	945.666745
CTNS	0.001057455	945.666745
ALG1	0.000705219	1418
MTTP	0.000705219	1418
GALNS	0.000705219	1418
MAN2B1	0.000705094	1418.250044
PC	0.000705094	1418.250044
EIF2B5	0.000705094	1418.250044
SLC7A7	0.000705094	1418.250044
ALG6	0.000705094	1418.250044
ETHE1	0.000705094	1418.250044
PEX6	0.000705094	1418.250044
ABCB11	0.000705094	1418.250044
NCF2	0.000705094	1418.250044
POLG	0.000705094	1418.250044
NBN	0.000705094	1418.250044
ETFA	0.000705094	1418.250044
NAGLU	0.000705094	1418.250044
GBE1	0.000705094	1418.250044
USH1C	0.000705094	1418.250044
MFSD8	0.000705094	1418.250044
TPP1	0.000705094	1418.250044
BCKDHA	0.000705094	1418.250044
AGA	0.000352609	2836
SLC25A15	0.000352609	2836
HMGCL	0.000352609	2836
HADHA	0.000352609	2836
ATP8B1	0.000352609	2836
COLQ	0.000352609	2836
GLDC	0.000352609	2836
NPC2	0.000352609	2836
AMT	0.000352609	2836
DLD	0.000352609	2836
SLC26A2	0.000352609	2836
ABCA12	0.000352609	2836
DBT	0.000352609	2836
GNS	0.000352609	2836
PROP1	0.000352609	2836
ATM	0.000352609	2836
CLN6	0.000352609	2836
SGCG	0.000352609	2836
SGCA	0.000352609	2836
ASL	0.000352609	2836
ETFB	0.000352609	2836
QDPR	0.000352609	2836
BBS1	0.000352609	2836
MPI	0.000352609	2836

FAH	0.000352609	2836
TTPA	0.000352609	2836
MCOLN1	0.000352609	2836
CHRNE	0.000352609	2836
DOLK	0	Inf
CLN8	0	Inf
LHX3	0	Inf
GHR	0	Inf
PPT1	0	Inf
CTSD	0	Inf
MMAB	0	Inf
G6PC3	0	Inf
LARGE1	0	Inf
SUMF1	0	Inf
B4GALT1	0	Inf
COL1A2	0	Inf
PEX7	0	Inf
GCH1	0	Inf
SLC35C1	0	Inf
NCF1	0	Inf
SGCB	0	Inf
CLN3	0	Inf
CHRNA1	0	Inf
SLC17A5	0	Inf
ARG1	0	Inf
USH1G	0	Inf
DPAGT1	0	Inf
CLRN1	0	Inf
CPT1A	0	Inf
KCNJ11	0	Inf
HBA1	0	Inf
POU1F1	0	Inf
